# Retinal endothelial cell phenotypic modifications during experimental autoimmune uveitis: a transcriptomic approach

**DOI:** 10.1186/s12886-020-1333-5

**Published:** 2020-03-17

**Authors:** Deborah A. Lipski, Vincent Foucart, Rémi Dewispelaere, Laure E. Caspers, Matthieu Defrance, Catherine Bruyns, François Willermain

**Affiliations:** 1 Ophthalmology Group, IRIBHM (Institut de Recherche Interdisciplinaire en Biologie Humaine et Moléculaire), Université Libre de Bruxelles (ULB), Erasme Campus, Building C, Room C6.117, 808 Route de Lennik, 1070 Brussels, Belgium; 2 Ophthalmology Department of Erasme Hospital, Université Libre de Bruxelles (ULB), 808 Route de Lennik, 1070 Brussels, Belgium; 3 Ophthalmology Department of CHU Saint-Pierre, 322 Rue Haute, 1000 Brussels, Belgium; 4 Ophthalmology Department of CHU Brugmann, 4 Place Van Gehuchten, 1020 Brussels, Belgium; 5 Interuniversity Institute of Bioinformatics in Brussels, Université Libre de Bruxelles - Vrije Universiteit Brussel, La Plaine Campus, BC building, 6th floor, CP 263, Triomflaan, 1050 Brussels, Belgium

**Keywords:** Uveitis, Inflammation, Blood-retinal barrier, RNA-Seq, Transcriptome, Endothelial cells, serpina3n, Lipocalin 2, ackr1, lrg1

## Abstract

**Background:**

Blood-retinal barrier cells are known to exhibit a massive phenotypic change during experimental autoimmune uveitis (EAU) development. In an attempt to investigate the mechanisms of blood-retinal barrier (BRB) breakdown at a global level, we studied the gene regulation of total retinal cells and retinal endothelial cells during non-infectious uveitis.

**Methods:**

Retinal endothelial cells were isolated by flow cytometry either in Tie2-GFP mice (CD31^+^ CD45^−^ GFP^+^ cells), or in wild type C57BL/6 mice (CD31^+^ CD45^−^ endoglin^+^ cells). EAU was induced in C57BL/6 mice by adoptive transfer of IRBP1–20-specific T cells. Total retinal cells and retinal endothelial cells from naïve and EAU mice were sorted and their gene expression compared by RNA-Seq. Protein expression of selected genes was validated by immunofluorescence on retinal wholemounts and cryosections and by flow cytometry.

**Results:**

Retinal endothelial cell sorting in wild type C57BL/6 mice was validated by comparative transcriptome analysis with retinal endothelial cells sorted from Tie2-GFP mice, which express GFP under the control of the endothelial-specific receptor tyrosine kinase promoter Tie2. RNA-Seq analysis of total retinal cells mainly brought to light upregulation of genes involved in antigen presentation and T cell activation during EAU. Specific transcriptome analysis of retinal endothelial cells allowed us to identify 82 genes modulated in retinal endothelial cells during EAU development. Protein expression of 5 of those genes (serpina3n, lcn2, ackr1, lrg1 and lamc3) was validated at the level of inner BRB cells.

**Conclusion:**

Those data not only confirm the involvement of known pathogenic molecules but further provide a list of new candidate genes and pathways possibly implicated in inner BRB breakdown during non-infectious posterior uveitis.

## Background

In physiological conditions, the eye is isolated from systemic circulation by an anatomical barrier that restricts the entry of potentially toxic molecules, pathogens and immune cells into the eye. The blood-retinal barrier (BRB) is composed of 2 distinct parts. On the one side, the outer BRB is formed by tight junctions between retinal pigment epithelial (RPE) cells, separating fenestrated choroidal vessels from the outer retina. On the other side, the inner BRB is formed by tightly sealed vascular non-fenestrated endothelial cells (ECs), surrounded by pericytes and supported by Müller cell and astrocyte foot processes. Similarly to the blood-brain barrier (BBB) in the central nervous system, many of the properties of the BRB are manifested in ECs [1].

Alteration of BRB properties is a hallmark of ocular inflammatory diseases. Although BRB breakdown may be beneficial, allowing immune cells to clear debris and repair damages, it can also be harmful, leading to edema by accumulation of water and plasma proteins, tissue damage by inflammatory cells and ultimately vision loss. During non-infectious posterior uveitis, activated T cells produce cytokines and chemokines that activate retinal vessels [2], resulting in a drastic change of retinal EC phenotype, comprising among others upregulation of adhesion molecules such as P selectin, ICAM-1 [3] and VCAM-1 [4] and downregulation of tight junction proteins [5]. This leads to the recruitment of a wide range of circulating leukocytes such as monocytes/macrophages, granulocytes, NK cells, NKT cells and γδ T cells that are directly responsible for tissue damage [2]. The BRB then becomes increasingly permeable, allowing more immune cells into the eye, creating an amplification loop of the pathogenic process.

Although some individual alterations contributing to BRB dysfunction have been described, the underlying molecular mechanisms are still largely unknown. A transcriptomic study of total retinal cells and retinal ECs during experimental autoimmune uveitis (EAU) would thus represent an interesting approach to bring to light new target genes and signaling pathways involved in BRB breakdown. In the past decade, a few genome wide expression data sets have been generated for retinal ECs [6–18] (Table 1). Gene profiling studies have already confirmed that ECs from the immune-privileged central nervous system have a particular gene signature, compared to ECs in other tissues [1]. A few transcriptomic studies have been performed in human retinal ECs [12, 15, 17, 19]. Compared to uveal ECs, retinal ECs were found to express higher levels of transcripts involved in the immune response, including cell adhesion molecules, cytokines, chemokines, receptors and enzymes participating in the synthesis of inflammatory proteins. This is consistent with their suspected role in the regulation of leukocyte trafficking and inflammatory reaction during uveitis [6, 12]. Furthermore, the expression of candidate genes such as adhesion molecules (e.g. ICAM-1, VCAM-1, E and P selectins) and chemokines (CXCL10, CCL20 and CX3CR1) was shown to be induced in cultured human ECs by inflammatory stimuli [19, 20]. However, as illustrated in Table 1**,** most studies were performed on cultured ECs, which rapidly lose their barrier properties [21]. Furthermore, although the effects of inflammatory stimuli by exposure to toxoplasma gondii, LPS or TNF-α [7, 15, 17, 18] on gene expression were explored in EC cultures, to the best of our knowledge no study has investigated yet how EC gene expression is affected in vivo during non-infectious uveitis.

**Table 1:** Recent genome wide expression data sets for retinal endothelial cells

Study	Method of retinal EC isolation	Method of gene expression analysis	Species	Other cells studied	Disease/ conditions
Zhao et al., 2016 [6]	Cultured human retinal ECs (HRECs)	miRNA microarray	Human	/	Exposure to normal glucose or high glucose
Savage et al., 2015 [7]	Cultured human retinal microvascular ECs (HRMEC)	RNA-Seq	Human	/	Exposure to TNF-α in the presence or absence of the NFAT-specific inhibitor INCA-6 vs vehicle-treated control
Wang et al., 2013 [8]	Freshly isolated mouse microvessels (CD31-based magnetic purification)	Microarray	Mouse	/	Cells from *rd1, Vldlr*^*−/−*^ and *Grhl3*^*ct*^/J curly tail mice (mutants with remodelling of the retinal vasculature) vs naive C75BL/6J
Kusuhara et al., 2012 [9]	FACS based on GFP expression in P8 Tie2GFP transgenic mice	Microarray	Mouse	GFP- retinal cells	/
Steinle et al., 2012 [10]	Cultured human retinal ECs	Microarray	Human	/	Melphalan or carboplatin treatment vs untreated cells
Takase et al., 2012 [11]	FACS based on GFP expression in Flk1^+^/GFP embryos	Microarray	Mouse	Whole WT and Flk1 KO embryos	/
Browning et al., 2011 [12]	Retinal EC cultures from 3 human donors (CD31-based magnetic purification)	Microarray	Human	Donor-matched iris and choroidal ECs + cultured human umbilical vein ECs (HUVEC)	/
Strasser et al., 2010 [13]	Laser capture microdissection of retinal endothelial tip cells and endothelial stalk cells 24-36h after birth	Microarray	Mouse	/	/
Abukawa et al., 2009 [14]	Conditionally immortalized rat retinal capillary ECs (TR-iBRB2)	Microarray	Rat	/	Incubation with Müller cell conditioned medium vs control
Smith et al., 2007 [15]	Separate primary retinal EC cultures from 6 human donors (CD31-based magnetic purification)	Microarray	Human	Donor-matched choroidal ECs	Exposure to Toxoplasma gondii tachyzoïtes or LPS vs control
Ohtsuki et al., 2005 [16]	Conditionally immortalized rat retinal capillary ECs (TR-iBRB2 and TR-iBRB9)	mRNA differential display analysis	Rat	Brain capillary ECs (TR-BBB)	/
Silverman et al., 2005 [17]	Separate retinal EC cultures from 4 human donors (CD31-based magnetic purification)	Microarray	Human	Donor-matched iris ECs	Exposure to LPS or TNF-α vs control
Knight et al., 2005 [18]	Immortalized rat retinal vascular ECs (SV40 large T immortalized cell line JG2/1)	Microarray	Rat	/	Exposure to Toxoplasma gondii vs control

Pubmed search conducted with the terms transcriptome/ RNA-Seq/ microarray/ genome wide AND endothelial/ endothelium/ vascular AND retina

In this work, we have decided to analyze the transcriptome of freshly isolated total retinal cells and retinal ECs from EAU and naïve retinas used as control. Using RNA-Seq, we identified a series of genes that are modulated during EAU development. The expression of some of those genes was then analyzed by immunofluorescence (IF) and flow cytometry.

## Methods

### Reagents and animals

Interphotoreceptor retinoid-binding peptide (IRBP) 1–20 (GPTHLFQPSLVLDMAKVLLD), representing residues 1–20 of human IRBP, was synthesized by New England Peptide (Gardner, MA., USA). Pertussis toxin (PTX) and complete Freund’s adjuvant (CFA) were purchased from Sigma-Aldrich (Bornem, Belgium). Pathogen-free female C57BL/6 J mice (6- to 10-weeks old), purchased from Janvier (Genest St Isle, France) were housed at the animal facilities in accordance with European guidelines. FVB/N homozygous Tie2-GFP transgenic male mice (Tg(TIE2GFP)287Sato/J, stock number 003658), which express green fluorescent protein (GFP) under the control of the endothelial-specific receptor tyrosine kinase Tie2 (or Tek) promoter, were purchased from Jackson Laboratories (Bar Harbor, ME). Animal treatment conformed to the ARVO Statement for the Use of Animals in Ophthalmic and Vision Research. All cells were cultured in RPMI 1640 medium supplemented with 25 mM HEPES, 10% fetal bovine serum, 1% L-glutamine, 1% sodium-pyruvate, 100 IU/ml penicillin, 100 g/ml streptomycin, 5.10^− 5^ M β-mercaptoethanol in a 5% CO2 and 95% humidity incubator.

### Backcrossing and genotyping

Two FVB/N Tie2-GFP males (Tg(TIE2GFP)287Sato/J, stock number 003658) were paired with wild type (WT) C57BL/6 J female mice. C57BL/6 J-Tie2-GFP mice were then generated by backcrossing genetically selected Tie2-GFP carrier males with WT C57BL/6 J females over 10 generations. The resulting litters from each pairing were genotyped by standard PCR amplification of the transgene on genomic DNA extracted from tail tissue samples, as suggested by Jackson Laboratories.

### Adoptive transfer model of experimental autoimmune uveitis

EAU was induced by adoptive transfer (AT) of autoreactive lymphocytes following the protocol of Shao H et al. [22]. Briefly, naive C57BL/6 J mice were immunized with a subcutaneous injection in each hind leg of 50 μl of a mixture containing 500 μg/100 μl IRBP peptide 1–20 in a 1:1 emulsion of CFA enriched with 2.5 mg/ml of heat-inactivated *Mycobacterium tuberculosis*. All animals received simultaneously an intraperitoneal injection of 1 μg of PTX. Twelve days after immunization, mice were euthanized by cervical dislocation and their spleen and draining lymph nodes dissected and dissociated. Spleen cell suspensions were enriched in T lymphocytes through passage on nylon wool fiber columns, then pooled with total lymph node cells and re-stimulated in vitro with IRBP1–20 (1 μg/ml). After 2 days in culture, cells were injected intraperitoneally into naive C57BL/6 J mice (5 × 10^6^ cells/mouse).

### Disease grading

A clinical grading was performed 21 days after AT. Mice were anaesthetized by a 50 μl intraperitoneal injection of a Rompun (0,2%) and Ketalar (20 mg/ml) mixture. Pupils were dilated with tropicamid (5 mg/ml) and phenylephrine (1.5 mg/ml) and eyes were examined under the slit-lamp of a surgical microscope (Zeiss, Göttingen, Germany) by using a cover slip coated in a viscoelastic gel (synthetic polymer of acrylic acid 2 mg/g, Vidisic, Tramedico, Belgium) and positioned on the cornea. The clinical grading was performed independently by two ophthalmologists, based on a system adapted from Xu et al. [23]. Briefly, vitritis, optic neuropathy, retinitis and vasculitis were separately scored in each eye, from 0 (no disease) to 4 (highly severe disease) with half point increments and averaged to generate the clinical score of the eye on a scale from 1 to 4. The clinical score attributed to one mouse corresponds to the mean of the scores of the 2 eyes. The animals were euthanized by cervical dislocation.

### Immunohistology

#### Immunofluorescence stainings on retinal cryosections

At day 21 after disease induction, mice were euthanized by cervical dislocation while still anaesthetized and unconscious (see section 4.4). Eyes were collected, prefixed for 6 h at 4 °C in PFA (paraformaldehyde) 4%, sucrose 3% and then put in three successive baths containing 5, 10 and 18% sucrose in phosphate-buffered saline (PBS), respectively, for 24 h each. Entire eyes were embedded in OCT medium (Sakura, Antwerp, Belgium) and cut in 16 μm-thick frozen sections using a cryostat (CM3050S Leica). The MOM (mouse-on-mouse) Basic Kit (Vector Laboratories, Labconsult, Brussels) was used to prevent high background staining. Cryosections were fixed with PFA 4% for 15 min and blocked in TBS (Tris 10 mM, NaCl 0.9%, pH 7.6) supplemented with MOM IgG blocking solution and Triton 0.3% for 2 h. Sections were incubated overnight with the following primary antibodies, alone or in different combinations as indicated in results: anti-endoglin (goat, 1/200; BD Biosciences), anti-CD31 (rat, 1/200, BD Biosciences)*,* anti-lrg1 (rabbit, 1/100, Proteintech, Manchester), anti-serpina3n (goat, 1/200, R&D systems), anti-lcn2 (goat, R&D systems), anti-lamC3 (1/10000, generous gift from W. J. Brunken) and anti-ackr1 (1/2000, generous gift from U. von Andrian) and diluted in TBS supplemented with MOM kit protein concentrate. After three washings in TBS, the sections were incubated in the dark for 1 h30 with species-specific secondary antibodies coupled to different fluorochromes, as indicated in data, then with Hoechst to stain the nuclei (Invitrogen, Gent, Belgium). After several washings, sections were mounted in Glycergel (Dako, Agilent Technologies, Diegem, Belgium) supplemented with 2.5% Dabco (Sigma-Aldrich). Pictures of immunostainings were acquired using an AxioImager Z1 microscope equipped with an AxioCamMR camera (Carl Zeiss, Inc.) and the z-stack mode of the Axiovision acquisition software. Z-stacks were processed using the Imaris deconvolution software.

#### Immunofluorescence stainings on retinal wholemount preparations

At day 21 after disease induction, mice were euthanized by cervical dislocation while still anaesthetized and unconscious (see section 4.4). Eyes were collected and immediately immersed in PFA 4% for 1 h at 4 °C. Eyes were then dissected in ice-cold PBS: the anterior segment of the globe, crystalline lens and vitreous were removed and the retina was carefully peeled from the RPE. Whole retinas were fixed in 70% ethanol for 1 h, rinsed 3 times in PBS (10 min each), blocked with a solution containing 3% milk and 3% bovine serum albumin (BSA) in PBS for 1 h and incubated with different combinations of primary antibodies in a PBS-BSA1% solution overnight at room temperature. Retinas were rinsed 3 times in PBS (20 min each) and incubated sequentially for 1 h with each secondary antibody. After 3 final washings in PBS (20 min each), the retinas were flattened by radial incisions and mounted with Vectashield antifade mounting medium with DAPI (Labconsult, Brussels).

### Preparation of retinal single cell suspensions

At day 21 after disease induction, mice were euthanized by cervical dislocation while still anaesthetized and unconscious (see section 4.4), and the eyes immediately enucleated. Naïve eyes were used as control. Eyes were carefully hemisected in Hank’s Balanced Salt Solution (HBSS) buffer containing penicillin/streptomycin 1%, with surgical scissors under a surgical microscope. Retinal tissue was isolated and rinsed in HBSS buffer. The 2 retinas of each mouse were pooled and cut into small pieces before enzymatic digestion in 3 ml HBSS containing 1,6 mg/ml Liberase DL (Roche, Vilvoorde, Belgium) and 0,1 mg/ml DNase I (Sigma-Aldrich) at 37 °C for 45 min. Cell dissociation was stimulated by pipetting every 15 min. Cells were washed with Dulbecco modified eagle medium (DMEM)/10% fetal bovine serum (FBS) and filtered through a 40-μm cell strainer to obtain a single cell suspension [24]. The yield was approximately 2 to 3 million retinal cells per mouse.

### Fluorescence-activated cell sorting (FACS) analysis

Retinal single cell suspensions were tested by flow cytometry for the expression of GFP, endoglin (CD105, endothelial cell marker), CD31 (PECAM-1, endothelial cell marker), CD45 (pan-leukocyte marker), ackr1, lrg1 and lamc3 using specific antibodies (BD Biosciences) coupled to different fluorochromes, except for GFP whose native fluorescence was observed. Cells were incubated with the relevant antibodies for 20 min at 4 °C, washed and re-suspended in FACS buffer. Live cells were gated with Hoechst 1/4000 and debris and doublets were excluded. Up to one million total cells per sample were analyzed on an LSR-Fortessa flow cytometer using the CellQuest Software (BD Biosciences). Isotypes and *Fluorescence minus one* (FMO) controls were used for accurate gating. Compensations were performed using BD CompBeads (BD Biosciences).

### Analysis of retinal cell gene expression

#### Purification of total retinal cells and retinal endothelial cells

Three weeks after AT, mice were sacrificed, and retinal single cell suspensions prepared as described above. Naïve eyes were used as controls. Cells were stained with APC-labeled anti-CD31, PECy7-labeled anti-CD45 and when applicable PE-labeled anti-endoglin antibodies. Total live retinal cells (DAPI-) and then retinal ECs (either GFP + CD31 + CD45- or endoglin+CD31 + CD45- cells) were separately sorted by preparative flow cytometry using a FACSAria with the FACSDiva Software (BD). Due to the low cell number obtained from each mouse, at least 3 mice were pooled to generate each EAU sample and at least 2 mice for naïve samples. Cells were sorted directly in lysis buffer, vortexed for 30 s and flash frozen in liquid nitrogen.

#### RNA extraction

RNA extraction was performed using the MiRNeasy MicroKit (Qiagen) according to the manufacturer’s recommendations and a DNase step to avoid DNA contamination. RNA quality was assessed using the Agilent 2100 Bioanalyzer with RNA 6000 Pico kit (Agilent Technologies).

#### RNA processing and RNA sequencing

Indexed cDNA libraries were prepared using the Ovation Single Cell RNA-Seq system (Nugen) (for samples isolated from transgenic mice) or the Ovation SoLo RNAseq system (Nugen) (for samples isolated from WT mice, since the Ovation Single Cell RNA-Seq system was then no longer available from Nugen). The multiplexed libraries were loaded, and sequences were produced using a TruSeq PE cluster and SBS-kit on a HiSeq 1500 (Illumina). Approximately 25 million paired-end reads/sample were mapped against the mouse reference genome (NCBI Build 37/UCSC mm9) using STAR software to generate read alignments for each sample. Expression levels were quantified using the featureCounts [25] tool and the UCSC RefSeq gene annotation as a reference (exons only, genes as meta features). Differential analysis between groups was performed using the EdgeR package (quasi-likelihood F-tests). Normalized expression levels were estimated using the EdgeR rpm function and converted to log_2_ FPKM (fragments per kilobase of exon per million mapped reads) after resetting low FPKMs to 1. To perform blind clustering analysis, genes were selected based on the overall variance between samples (independently of their category), by keeping only the 120 most variant ones. Functional analysis was performed using the Database for Annotation, Visualization, and Integrated Discovery (DAVID) web-based functional annotation tool [26].

## Results

In an attempt to investigate the local mechanisms of non-infectious posterior uveitis development at a global level, we aimed to study the gene regulation of total retinal cells and more specifically retinal ECs during EAU.

We first set up a method to purify and gene profile BRB-forming retinal ECs. We then compared the transcriptional profile of total retinal cells during AT EAU to that of naïve mice and finally performed the same analysis for retinal ECs.

### Purification and gene profiling of naïve retinal endothelial cells

The first challenge to be addressed pertained to the isolation of retinal ECs, which represent a small percentage of cells to extract from an already very small tissue.

**Fig. 1:**
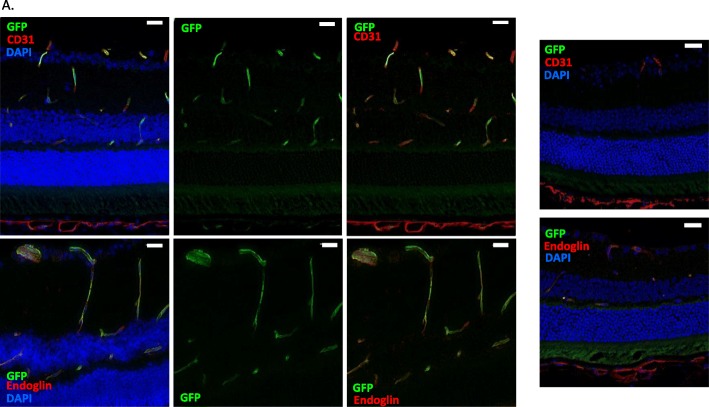
GFP is expressed by endothelial cells in heterozygous Tie2-GFP FVB/N-C57BL/6 J mice. *Immunofluorescence analysis*: eye cryosections of heterozygous Tie2-GFP FVB/N-C57BL/6 naïve mice (*left*) were prepared and stained for GFP (green) and either CD31 (*top*) or endoglin (*bottom*) (red) detection. WT C57BL/6 naive eyes were used as control (*right)*. Cell nuclei were stained with Hoechst (blue). Each picture was chosen as representative of an experiment conducted on 3 or more animals. Scale bars indicate 20 μm

As a first approach to retinal EC sorting, we decided to use transgenic Tie2-GFP mice, which express GFP under the control of the pan-endothelial Tie2 promoter (transgenic strategy). However, FVB/N-Tie2-GFP mice are not susceptible to EAU induction, as shown in Additional file 1: Fig. S1. We thus generated C57BL/6 J-Tie2-GFP mice by crossing founder FVB/N-Tie2-GFP males with WT C57BL/6 J mice and subsequently backcrossing genetically selected Tie2-GFP carrier males with WT C57BL/6 J females over 10 generations. In order to validate the specificity of GFP expression in retinal ECs, we first analyzed comparatively the expression of GFP with that of CD31 and endoglin (2 EC markers) in the retina of naïve heterozygous Tie2-GFP mice by immunofluorescence (IF) on eye cryosections. Figure 1 shows that the expression of GFP matches that of CD31 and endoglin in the retina of naive heterozygous Tie2-GFP mice. Unfortunately, we unexpectedly observed an extremely low transmission rate of the Tie2-GFP allele with successive backcrossing generations (Additional file 2: Fig. S2). Indeed, while 100% of F1 mice were carriers, the proportion dropped to 0% for F10 mice.

**Fig. 2:**
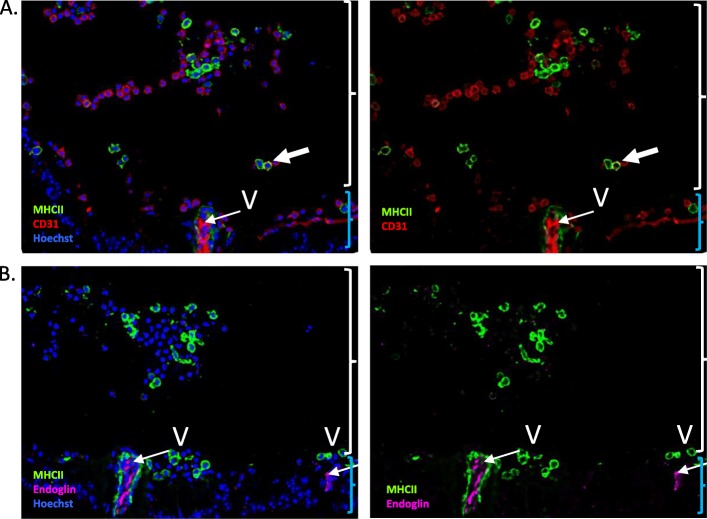
Expression of endothelial cell markers CD31 and endoglin in the retina during experimental autoimmune uveitis. Three weeks after adoptive transfer, eye cryosections were prepared and stained for MHC Class II (used as disease marker, green) and CD31 (red) or endoglin (magenta) detection. Cell nuclei were stained with Hoechst (blue). Each picture was chosen as representative of an experiment conducted on 3 or more animals. **a**. CD31 expression in the retina during EAU. **b**. Endoglin expression in the retina during EAU. Thin arrows point to endothelial expression, thick arrows identify non-endothelial expression. “V” indicates vasculitis lesions. White curly brackets identify the vitreous, blue curly brackets identify the inner retina

We thus developed an alternative EC sorting strategy in C57BL/6 J WT mice, based on combined expression of the 2 retinal EC markers, CD31 and endoglin, that were previously tested by IF in Fig. 1 (WT strategy). We first analyzed CD31 and endoglin expression on AT EAU eye cryosections from WT C57BL/6 J mice by IF (Fig. 2). During EAU, retinal vessels are well identified by CD31 and endoglin stainings (Fig. 2**,** thin arrows). However, some cells infiltrating the retina and vitreous show CD31 expression, which is clearly not endothelial (Fig. 2**,** thick arrows), while endoglin expression seems to remain purely endothelial during EAU. This indicates a lack of specificity of CD31 expression to firmly identify retinal ECs, enhancing the necessity to combine different markers for accurate retinal EC sorting.

Healthy murine retinas were then processed into single cell suspensions by enzymatic digestion. ECs were further sorted by FACS relying on either the expression of GFP and CD31 (transgenic strategy) or the expression of CD31 and endoglin (WT strategy), both combined with the absence of CD45 expression. We have indeed detected, as previously reported in the literature, a few cells co-expressing GFP, CD31 and CD45 which may enlighten the presence of Tie2-expressing hematopoietic cells (data not shown) [27, 28]. Each sample was generated from a pool of at least 4 retinas from 2 naïve mice. As shown in Additional file 3: Fig. S3A, ECs represent about 0,1% of the retinal cell population (excluding dead cells, debris and doublets) in both strategies. The WT sorting strategy was tested on Tie2-GFP mice, as illustrated in Additional file 3: Fig. S3B. Among pre-gated CD31 + CD45- cells, the majority of cells express both endoglin and GFP. Among cells gated as *endothelial* with the WT strategy (endoglin+CD31 + CD45-, blue gate), the vast majority would also be selected as ECs with the transgenic sorting strategy (GFP + CD31 + CD45-, orange gate).

**Fig. 3:**
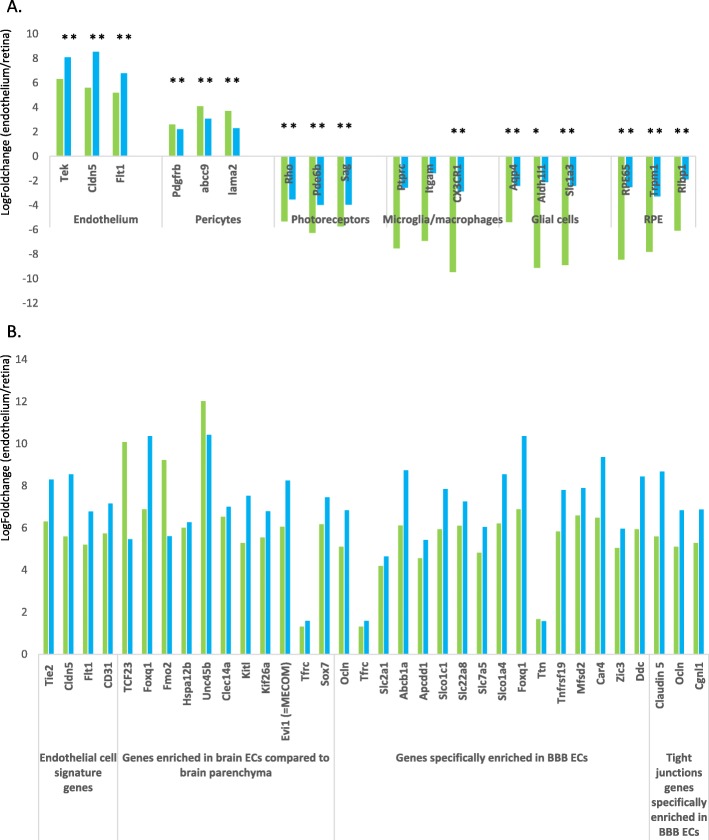
Endothelial cell markers are enriched in purified endothelial cells compared to total retina. **a**. *Purity of sorted endothelial cells*. RNA-Seq data were analyzed for the expression of endothelial, pericyte, photoreceptor, microglia/macrophage, glial and RPE cell markers to evaluate the purity of the endothelial cell samples. Each bar illustrates the relative expression of one marker by endothelial cells compared to total retinal cells (*LogFoldchange*). Green bars represent samples sorted with the transgenic strategy, blue bars represent samples sorted with the WT strategy. * FDR < 0,05, ** FDR < 0,01. **b**. *Enrichment of barrier-type endothelial cell markers in the endothelial cell samples*. RNA-Seq data were analyzed for the expression of endothelial cell (EC) markers previously identified as being specifically enriched in blood-brain barrier (BBB) ECs. Each bar illustrates the relative expression of one marker by ECs compared to total retinal cells (*LogFoldchange*). Green bars represent samples sorted with the transgenic strategy, blue bars represent samples sorted with the WT strategy. All represented FC reach statistical significance (FDR < 0,05)

We also compared the purity of the EC samples sorted with the 2 sorting strategies by RNA-Seq. For transcriptome studies, retinal ECs and total live retinal cells were separately sorted, both in C57BL/6 J-Tie2-GFP mice and in C57BL/6 J WT mice. Given the low expected number of cells, each sample was sorted from a pool of 3 mice. RNA was extracted and its quality tested before being processed for RNA-Seq analysis. RNA-Seq data were first analyzed for the expression of endothelial (Tie2, Cldn5, CD31), pericyte (Pdgfrb, Abcc9, Lama2), photoreceptor (Rho, Pde6b, SAg), microglia/macrophage (Ptprc (CD45), itgam (CD11b), Cx3cr1), macroglia (Aqp4, Aldh1l1, Slc1a3) and RPE (RPE65, Rgr, Rdh10) cell markers. Figure 3a illustrates comparative expression of those retinal cell markers by ECs versus total retina (green bars represent samples obtained with the transgenic strategy; blue bars represent samples obtained with the WT strategy). EC markers are highly enriched, and all non-endothelial markers are depleted in the EC fractions, whatever the sorting strategy, with the exception of pericyte markers. We next investigated the expression of more numerous EC markers, previously identified as specifically enriched in BBB ECs [1]. As illustrated in Fig. 3b, all those *barrier-type* EC markers are significantly enriched in the EC fractions, compared to total retina, with a similar pattern between the 2 sorting strategies.

### Analysis of retinal cell gene regulation during EAU

We then induced AT EAU in C57BL/6 J WT mice and sorted live total retinal cells and retinal ECs in order to identify regulated transcripts compared to naïve mice. Each sample was sorted from a pool of at least 3 mice. We analyzed a total of 5 AT EAU samples and 4 naïve samples.

The transcriptional profiles of replicates (samples of the same nature isolated from the same pool of mice), biological duplicates (samples of the same nature isolated from different pools of mice) and samples of different nature were compared, in order to identify the source of variation. As shown in Additional file 4: Fig. S4, the dispersion is lowest for replicates, intermediate for biological duplicates and highest for samples of different nature.

**Fig. 4:**
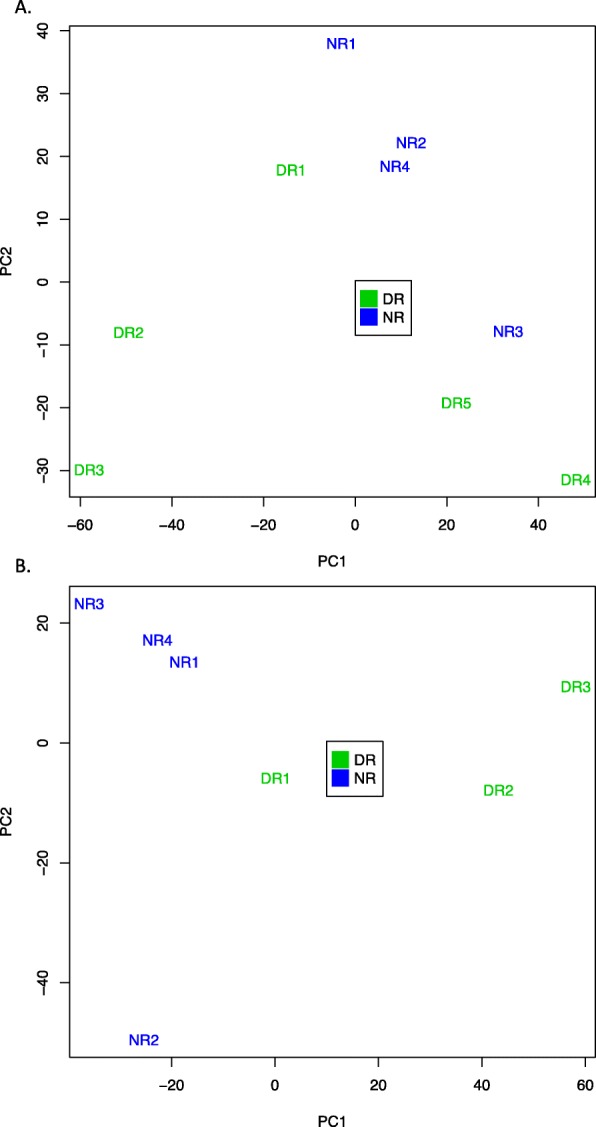
Principal component analysis of EAU and naive retinal cell samples. **a** PCA analysis of all EAU and naïve retinal cell samples (DR: diseased retina, NR: naive retina). **b** PCA analysis after elimination of 2 DR samples

A principal component analysis (PCA) was performed with all diseased and naïve retinal cell samples (Fig. 4a). PCA provides an unsupervised 2-dimensional graphic representation of the similarities and differences between samples. The PCA data show a tendency of diseased retinal cell samples (DR) to segregate from naïve retinal cell samples (NR). However, the transcriptome of 2 diseased retinal cell samples (DR4 and DR5) are closer to the cluster of naïve retinal cell samples. Those 2 samples are isolated from the only 2 pools of mice whose mean clinical grade was inferior to 2. When those 2 samples are eliminated, clearer segregation between diseased and naïve retinal cells is brought to light (Fig. 4b). Since the aim of the project was to target the differences between EAU and naïve cells, those 2 samples were dismissed for further analysis, which was thus performed on 3 diseased samples and 4 naïve samples.

Only genes with a Log fold change (FC) superior to 2 (absolute value) and a false detection rate (FDR) inferior to 0,05 were retained. With those filters, we identified 182 genes significantly regulated between diseased and naïve retinal cells, which were ranked according to their foldchange top down (Additional file 5: Fig. S5).

We then tried to classify those genes into families (Table 2).

**Table 2:**
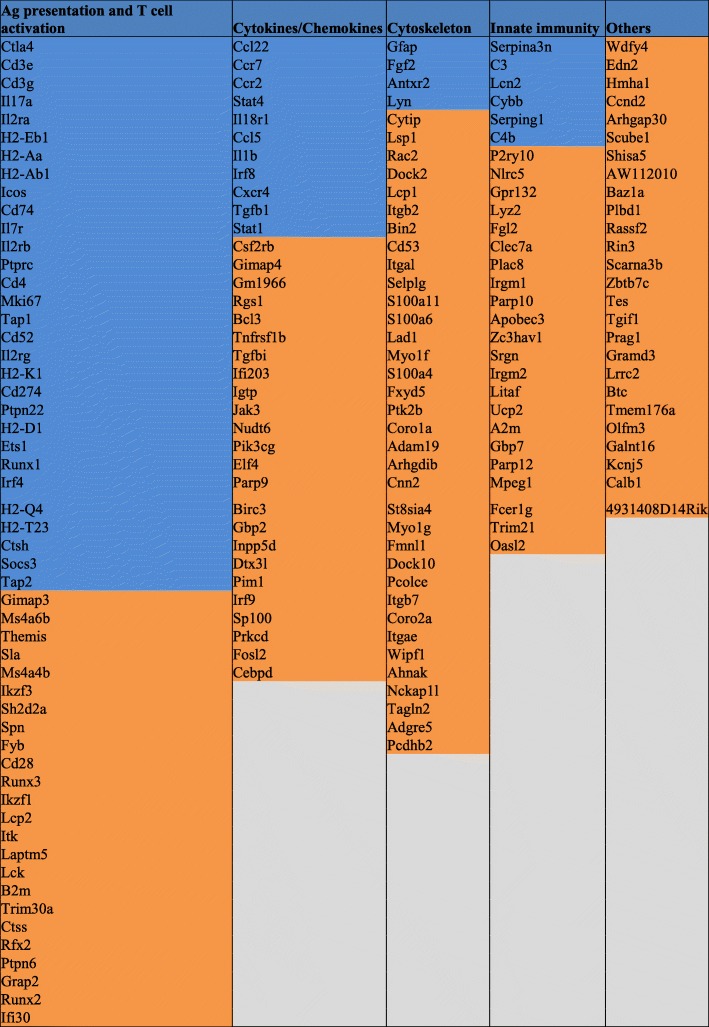
Classification of the 182 candidate genes into 4 groups

Legend: 182 genes were found to be significantly regulated between diseased and naïve retinal cells. Those genes were classified into 4 main groups. Genes whose function was previously described during uveitis appear in blue, genes whose function was not previously described in uveitis appear in orange

**Fig. 5:**
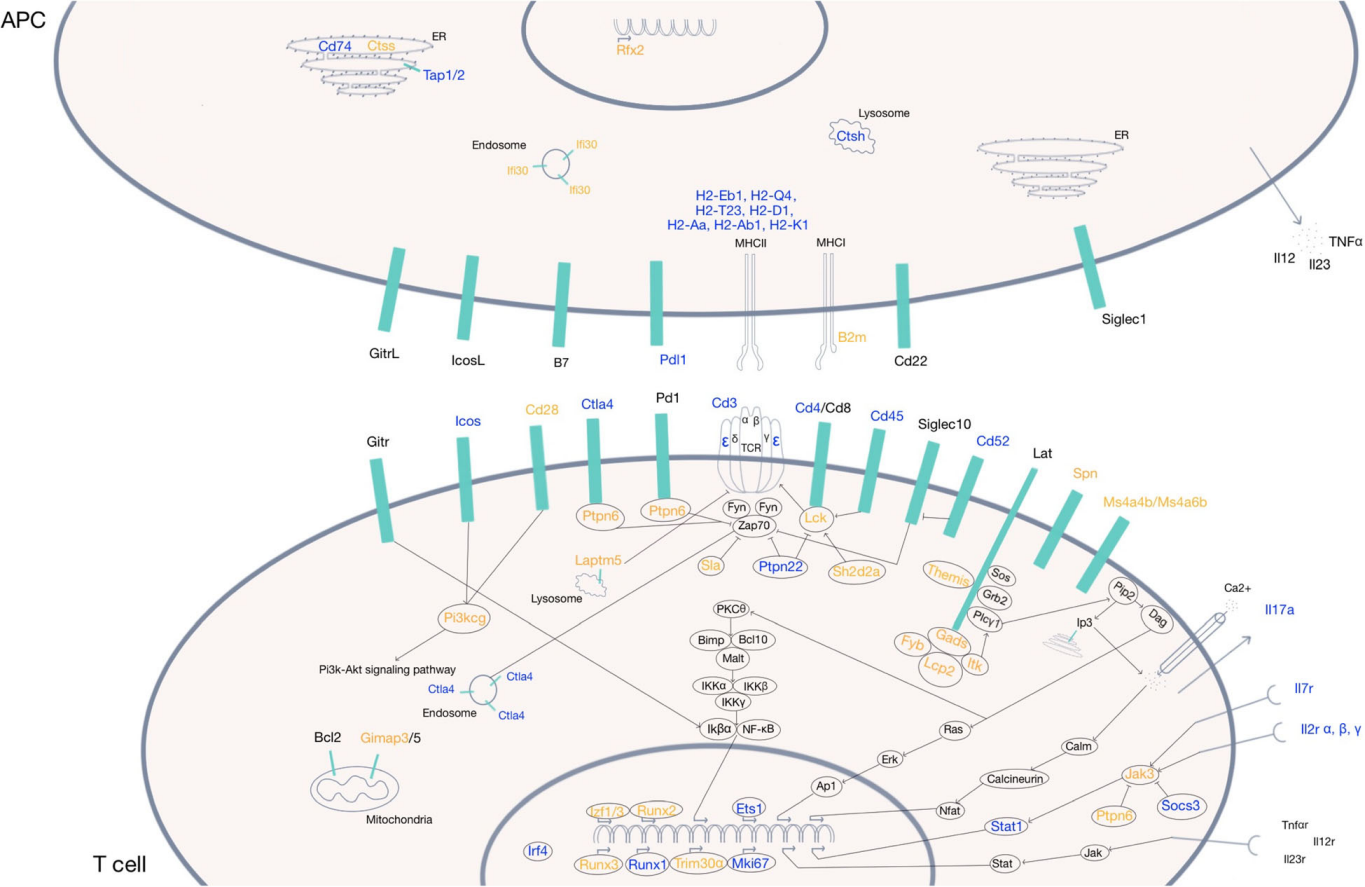
Schematic representation of the interaction between an antigen-presenting cell and a T cell. In orange: genes whose expression or function was not previously described in the context of uveitis. In blue: genes whose expression or function was previously described in the context of uveitis. In black: genes that do not belong to the list of 182 candidate genes

In order to identify the different pathways in which those genes are involved, we used DAVID, Pubmed and Genecards. Four main groups were identified: 52 genes playing a role in antigen presentation to T cells in association with MHC class II, 45 genes implicated in cytokine and chemokine signaling pathways, 39 genes taking part in cell mobility and 18 genes that contribute to innate immunity. However, 33 genes could not be attributed to one of these categories. Genes whose function was previously described during uveitis appear in blue, genes whose function was not previously described in uveitis appear in orange. Figure 5 graphically illustrates the role of the genes classified in group 1 (antigen presentation).

RNA Seq analysis of total retinal cells mainly brought to light upregulation of genes involved in antigen presentation and T cell activation during EAU. This approach thus reflects activation of local antigen-presenting cells and invasion of the retina by immunocompetent cells but does not allow specific detection of BRB cell gene modulation. This comforted us to adopt a more specific strategy targeting ECs.

### Identification of retinal endothelial cell gene regulation during EAU

Retinal ECs were isolated from the same EAU and naïve samples as total retinal cell samples, with the WT sorting strategy described above.

**Fig. 6:**
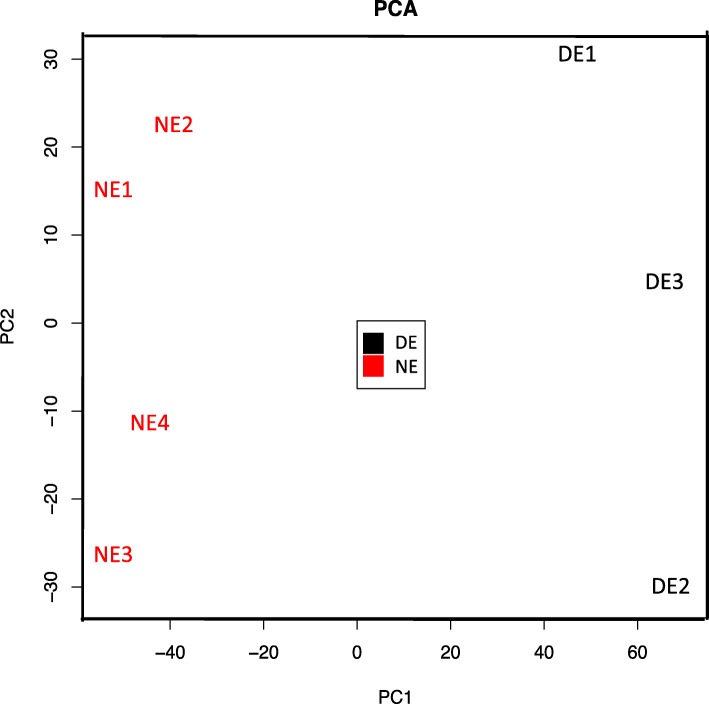
Principal component analysis shows segregation of EAU and naive endothelial cell samples. PCA analysis of endothelial, EAU and naive cell samples (DE: diseased endothelium, NE: naive endothelium)

A principal component analysis (PCA) was performed with EAU endothelial samples (DE, diseased endothelium) and naïve endothelial samples (NE, naïve endothelium) (Fig. 6). This PCA shows that DE and NE tend to cluster separately.

**Fig. 7:**
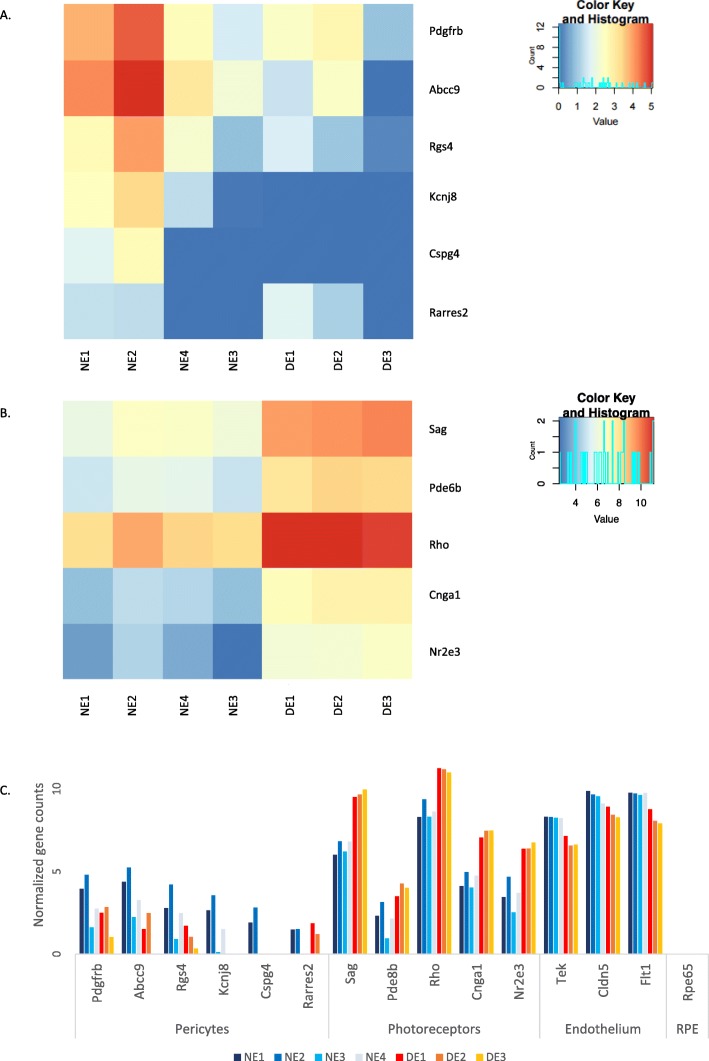
Identification of potential contaminants in EAU and naive retinal endothelial cell samples. **a** Heatmap illustrating comparative expression of pericyte markers by naive (NE) or diseased (DE) retinal endothelial cells. **b** Heatmap illustrating comparative expression of photoreceptor cell markers by naive (NE) or diseased (DE) retinal endothelial cells. **c** Histogram illustrating raw gene expression values for photoreceptor cell markers and pericyte markers by naive (NE) or diseased (DE) retinal endothelial cells. Normalized gene counts for endothelial cell markers and RPE marker RPE65 were added as reference

As illustrated in Additional file 6: Fig. S6, mRNA levels are accurately enriched or depleted for all markers used for cell sorting. As previously observed or mentioned in the literature, contamination of the transcriptome of EC samples by pericytes and photoreceptor genes was observed. Figure 7 indeed illustrates the expression of pericyte and photoreceptor cell markers by the different cell populations. The expression of pericyte markers is higher in NE samples, while expression of photoreceptor markers is predominant in DE samples.

**Fig. 8:**
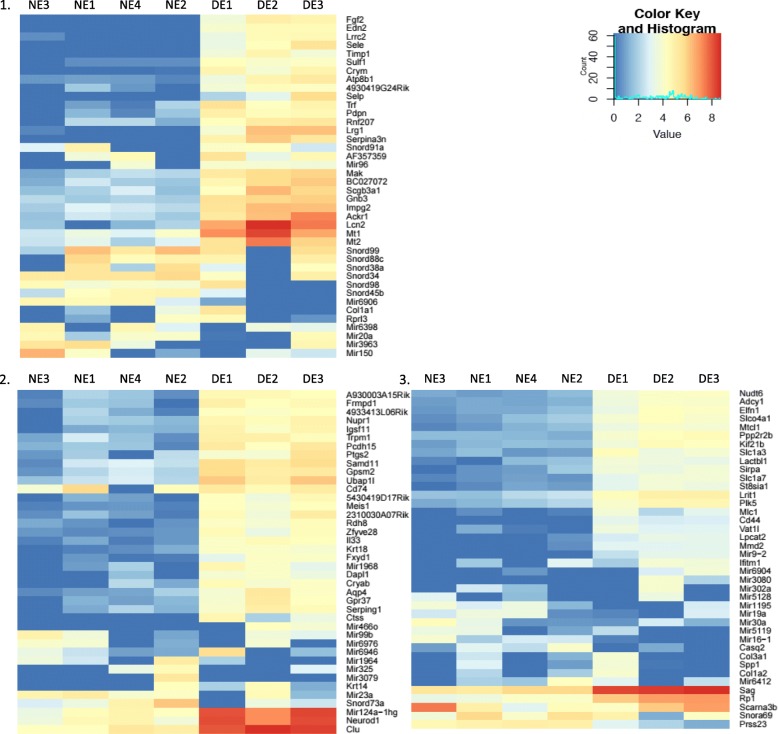
Analysis of most variant genes across EAU and naive retinal endothelial cell samples. Heatmaps illustrating the expression of the 120 most variant genes between DE and NE samples

**Fig. 9:**
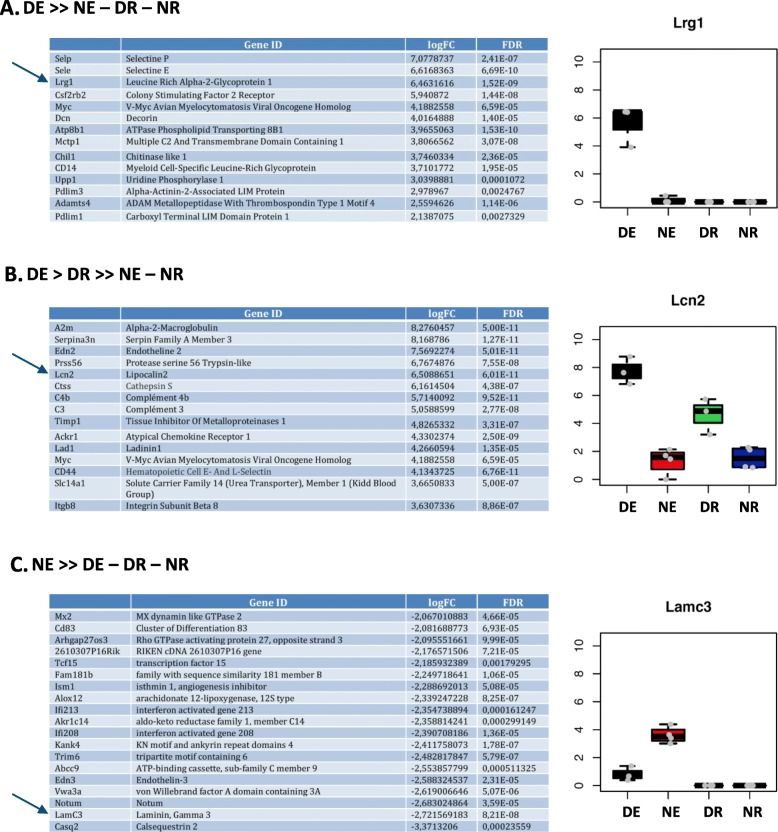
Selection of candidate genes based on their expression profile. Genes were first filtered with the bioinformatics formula DE vs NE + DE ≠ NR. Two profiles of expression were then selected, for which the risk of contamination by photoreceptor genes is minimal. **a**. The first profile corresponds to genes expressed only in DE (DE+, e.g. Lrg1). **b** The second profile corresponds to genes expressed in DE and in DR (DE+/DR+, e.g. Lcn2). **c** The third profile corresponds to genes whose expression is down-regulated in DE versus NE (DE-, e.g. Lamc3)

In order to select relevant genes, we first ranked by false detection rate (FDR) the list of significantly regulated genes (FDR < 0,05) between the 2 conditions. Unfortunately, as expected given the photoreceptor contamination, many of the highly regulated genes were photoreceptor genes, rendering the analysis tedious. In an attempt to bypass this issue, we then looked at the 120 most variant genes between DE and NE (Fig. 8). Among those 120 genes, 65 were eliminated because they did not reach statistical significance. In order to identify different profiles of gene expression, we selected a list of genes by using the bioinformatics formula ‘DE significantly different from NE AND DE ≠ NR’. Among those, we selected 3 expression profiles in which the expression in naïve retina is low, hoping to minimize any photoreceptor gene contamination. Those 3 profiles are illustrated in Fig. 9. The first profile corresponds to genes expressed only in DE (DE+). The second profile corresponds to genes expressed in DE and in DR (DE+/ DR+). The third profile corresponds to genes expressed in NE only (NE+). Among significantly regulated genes, we identified 14 genes with the DE+ profile, 15 genes with the DE+/DR+ profile and 19 genes with the NE+ profile.

Research in the literature was conducted for the most variant genes as well as for genes selected for their profile, in order to exclude genes associated with photoreceptors. 21 genes known to be expressed by photoreceptors, among which 6 genes implicated in the photo-transduction cascade, were eliminated (Additional file 7: Fig. S7). Finally, 82 candidate genes were retained, among which 10 were selected both through their variance and through their expression profile (Additional file 8: Fig. S8).

Functional analysis with the David web-based tool was performed with the list of candidate genes. A selection of gene ontology (GO) terms enriched in DE compared to NE samples is illustrated in Table 3, and includes items related to inflammation, cell adhesion, complement activation, extracellular matrix and angiogenesis.

**Table 3:** Identification of functions enriched in endothelial cells during EAU

Function	Fold enrichment (DE/NE)	*P*-value
Inflammatory response	6,2	8,0 × 10^− 4^
Innate immunity	6,0	2,8 × 10^− 2^
Cytokine activity	5,5	3,4 × 10^− 2^
Selectin superfamily	228,8	8,6 × 10^− 3^
Cell adhesion	3,8	1,9 × 10^− 2^
Leukocyte tethering or rolling	40,9	4,7 × 10^− 2^
Complement activation	40,9	4,7 × 10^− 2^
Extracellular matrix	4,5	5,8 × 10^− 2^
Positive regulation of angiogenesis	10,1	6,9 × 10^− 3^

legend: Functional analysis was performed with the Database for Annotation, Visualization and Integrated Discovery (DAVID) web-based tool on the list of identified candidate genes. The first column lists selected functions enriched in EAU endothelial samples, the second column shows fold enrichment and the third column the *p*-value

### Analysis of the expression of candidate gene products

The expression of many of the identified candidate genes was already validated in the literature, especially those with the expression profile ‘DE+’. Based on the availability of antibodies, we decided to analyze the expression of 5 of the most strongly regulated candidate genes at the protein level: serpin family A member 3 (serpina3n), lipocalin 2 (lcn2), atypical chemokine receptor 1 (ackr1), laminin γ3 (lamc3) and leucine rich alpha-2-glycoprotein 1 (lrg1). We looked at protein expression on both retinal cryosections and wholemount retinal preparations, as well as by flow cytometry when applicable.

**Fig. 10:**
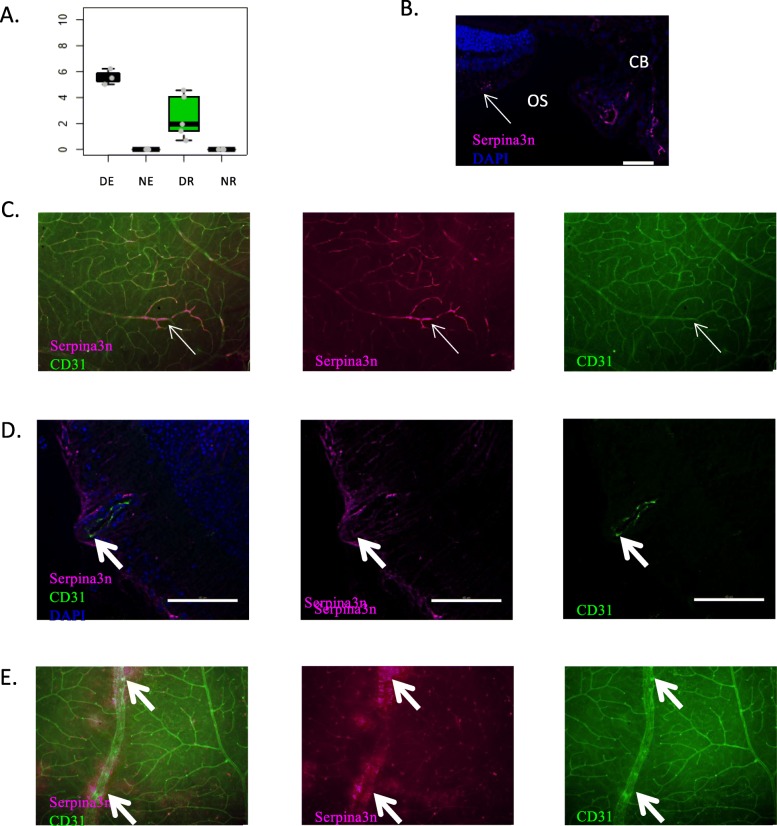
Analysis of serpina3n protein expression. Serpina3n expression was analyzed by immunofluorescence on retinal cryosections and retinal wholemounts 3 weeks after adoptive transfer. Naive eyes were used as control. Cryosections and wholemounts were prepared and stained for serpina3n (magenta) and CD31 (green) detection. Cell nuclei were stained with Hoechst (blue). **a**. Expression profile of Serpina3n at the mRNA level. **b** Analysis of serpina3n expression by immunofluorescence on naive retinal cryosections. CB = ciliary body, OS = ora serrata. **c** Analysis of serpina3n expression by immunofluorescence on naive retinal wholemounts. **d** Analysis of serpina3n expression by immunofluorescence on EAU retinal cryosections. **e** Analysis of serpina3n expression by immunofluorescence on EAU retinal wholemounts. Thin arrows point to serpina3n and CD31 co-staining in a normal vessel, thick arrows point to serpina3n and CD31 co-staining at the level of an inflamed vessel. Scale bars represent 20 μm

According to RNA-Seq data, serpina3n is expressed by both DE and DR (Fig. 10). Protein expression is visualized in naïve eye cryosections at the level of the ciliary body and very discretely on some optic nerve and retinal vessels (Fig. 10b). In naïve retinal wholemounts, discontinuous serpina3n expression is observed at the vascular level (Fig. 10c). Stronger expression develops in inflamed retinas, particularly at the level of vasculitis (thick arrows), both in ECs and perivascular glial cells (Fig. 10d). However, since expression is found both at the vascular and perivascular levels, it is difficult to differentiate purely endothelial expression from perivascular expression, e.g. by infiltrating immune cells. In EAU retinal wholemounts, serpina3n expression is found on most retinal vessels, co-localized with CD31 and is particularly intense at the level of inflamed vessels (thick arrows) (Fig. 10e).

**Fig. 11:**
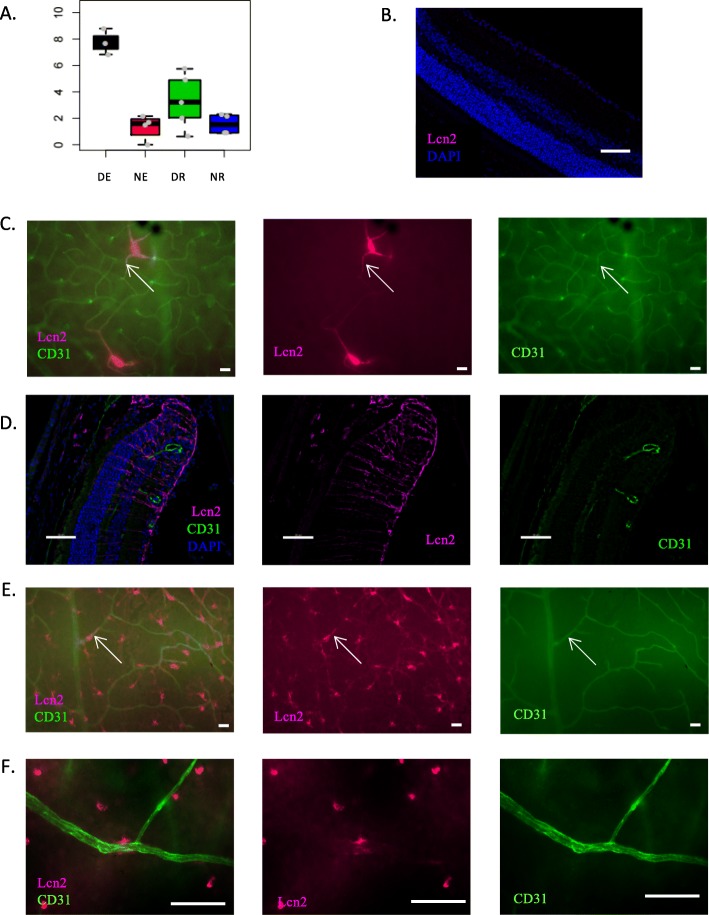
Analysis of lcn2 protein expression. Lcn2 expression was analyzed by immunofluorescence on retinal cryosections and retinal wholemounts 3 weeks after adoptive transfer. Naive eyes were used as control. Cryosections and wholemounts were prepared and stained for lcn2 (magenta) and CD31 (green) detection. Cell nuclei were stained with Hoechst (blue). **a** Expression profile of lcn2 at the mRNA level. **b** Analysis of lcn2 expression by immunofluorescence on naive retinal cryosections. **c** Analysis of lcn2 expression by immunofluorescence on naive retinal wholemounts. **d** Analysis of lcn2 expression by immunofluorescence on EAU retinal cryosections. **e** Analysis of lcn2 expression by immunofluorescence on EAU retinal wholemounts. **f** Analysis of lcn2 expression by immunofluorescence on EAU retinal wholemounts (× 40 objective). Arrows point to possible lcn2 and CD31 co-staining. Scale bars represent 20 μm

Lcn2 also has a DE+/DR+ expression profile (Fig. 11a). In naïve retinal cryosections, lcn2 expression is faintly present on the inner limiting membrane (Fig. 11b), while naïve retinal wholemounts show sparse lcn2 expression along rare retinal vessels (Fig. 11c, arrows). In EAU retinal cryosections, lcn2 staining has a macroglial aspect, particularly at the perivascular level. However, it does not seem to co-localize with CD31 (Fig. 11d). In EAU retinal wholemounts, although some co-staining with CD31 is observed (Fig. 11e**,** arrows), with a larger zoom lcn2 expression seems to be mostly perivascular (Fig. 11f**)**.

**Fig. 12:**
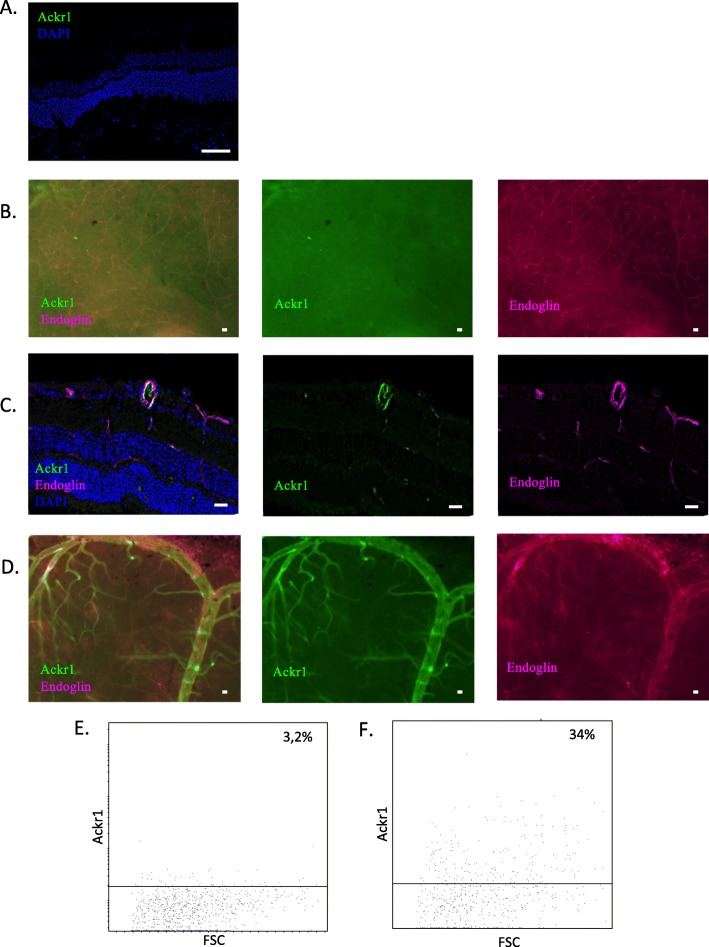
Analysis of ackr1 protein expression**.** Ackr1 expression was analyzed by immunofluorescence on retinal cryosections and retinal wholemounts 3 weeks after adoptive transfer, as well as by flow cytometry. Naive eyes were used as control. Cryosections and wholemounts were prepared and stained for ackr1 (red or green) and CD31 (magenta) detection. Cell nuclei were stained with Hoechst (blue). **a** Analysis of ackr1 expression by immunofluorescence on naive retinal cryosections. **b** Analysis of ackr1 expression by immunofluorescence on naive retinal wholemounts. **c** Analysis of ackr1 expression by immunofluorescence on EAU retinal cryosections. **d** Analysis of ackr1 expression by immunofluorescence on EAU retinal wholemounts. **e** FACS analysis of ackr1 expression by naïve retinal endothelial (CD31+ Endoglin+ CD45-) cells. **f** FACS analysis of ackr1 expression by EAU retinal endothelial (CD31+ Endoglin+ CD45-) cells. Scale bars represent 20 μm

Ackr1 belongs to the same DE+/DR+ profile. Ackr1 expression is purely endothelial and appears only in EAU cryosections (Fig. 12c) and wholemounts (Fig. 12d). This upregulation of ackr1 expression during EAU is confirmed by flow cytometry data (Fig. 12e and f). On average, 2% of naïve retinal ECs express ackr1, while 30,3% of retinal ECs express ackr1 during EAU. This upregulation is statistically significant (*n* = 3 independent pools of 3 mice, *p*-value for unpaired t-test: 0,0003).

**Fig. 13:**
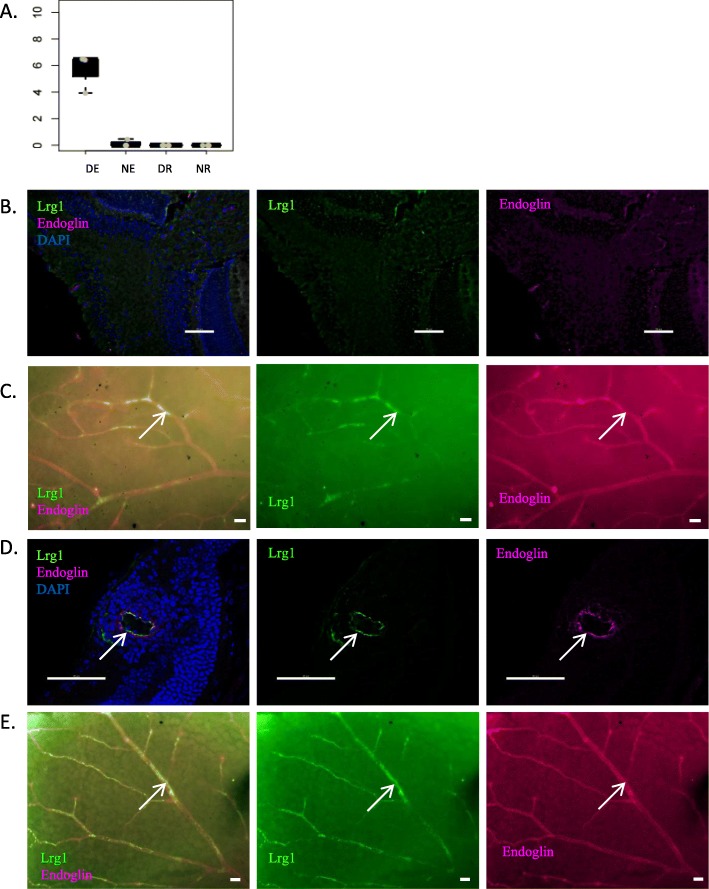
Analysis of lrg1 protein expression. Lrg1 expression was analyzed by immunofluorescence on retinal cryosections and retinal wholemounts 3 weeks after adoptive transfer. Naive eyes were used as control. Cryosections and wholemounts were prepared and stained for lrg1 (green) and endoglin (magenta) detection. Cell nuclei were stained with Hoechst (blue). **a** Expression profile of lrg1 at the mRNA level. **b** Analysis of lrg1 expression by immunofluorescence on naive retinal cryosections. **c** Analysis of lrg1 expression by immunofluorescence on naive retinal wholemounts. **d** Analysis of lrg1 expression by immunofluorescence on EAU retinal cryosections. **e** Analysis of lrg1 expression by immunofluorescence on EAU retinal wholemounts. Arrows point to lrg1 and endoglin co-staining. Scale bars represent 20 μm

According to RNA-Seq data, lrg1 is expressed only by DE (Fig. 13a). In naïve retinal sections, lrg1 is faintly expressed at the level of the ciliary body and at the ora serrata. No clear staining is observed in central retina (Fig. 13b). In naive retinal wholemounts, peripheral irregular expression of lrg1 appears at the vascular level, co-stained with CD31 (Fig. 13c). During uveitis, lrg1 upregulation is observed at the level of inflamed vessels on cryosections (Fig. 13d), while wholemounts show more diffuse lrg1 expression towards central retina than in naïve retina (Fig. 13e).

**Fig. 14:**
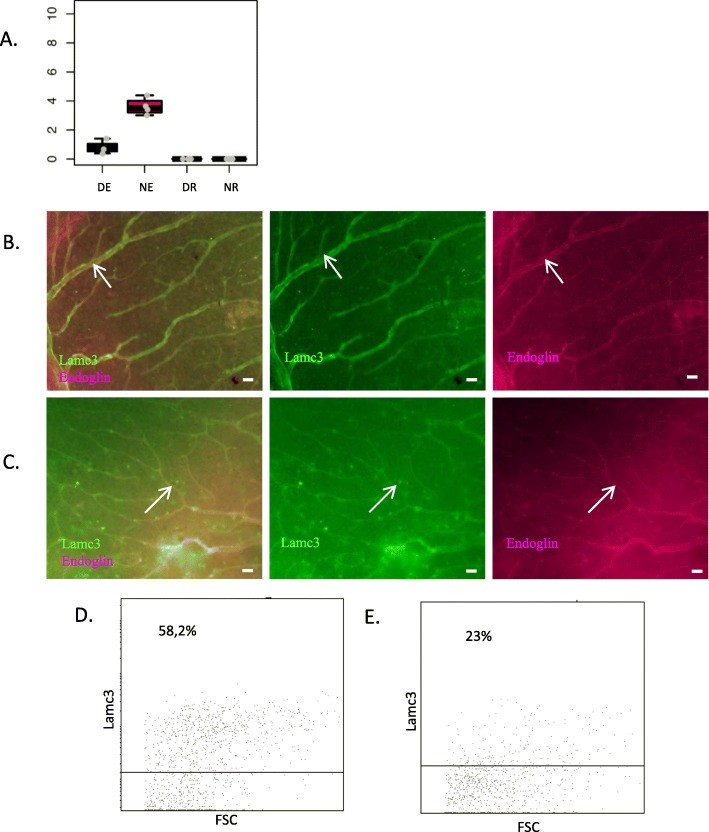
Analysis of Lamc3 protein expression. Lamc3 expression was analyzed by immunofluorescence on retinal cryosections and retinal wholemounts 3 weeks after adoptive transfer. Naive eyes were used as control. Cryosections and wholemounts were prepared and stained for Lamc3 (green) and endoglin (magenta) detection. Cell nuclei were stained with Hoechst (blue). **a** Expression profile of Lamc3 at the mRNA level. **b** Analysis of Lamc3 expression by immunofluorescence on naive retinal wholemounts. **c** Analysis of Lamc3 expression by immunofluorescence on EAU retinal wholemounts. **d** FACS analysis of Lamc3 expression by naïve retinal endothelial (CD31+ Endoglin+ CD45-) cells. **e** FACS analysis of Lamc3 expression by EAU retinal endothelial (CD31+ Endoglin+ CD45-) cells. Arrows point to possible Lamc3 and endoglin co-staining. Scale bars represent 20 μm

Lamc3 corresponds to the 3rd profile, with expression higher in NE than in DE (Fig. 14a). No clear difference is detected by IF on EAU versus naïve cryosections (data not shown). In retinal wholemounts, staining appears less organized and more dot-shaped in diseased retina (Fig. 14c) compared to naïve retina (Fig. 14b). However, flow cytometry data show a tendency of ECs to downregulate Lamc3 expression during EAU(Fig. 14d and e), which does not reach statistical significance (mean percentage of naïve retinal ECs expressing lamc3 45%, mean percentage of EAU retinal ECs expressing lamc3 30,7%, n = 3 independent pools of 3 mice, p-value for unpaired t-test 0,15).

## Discussion

The BRB allows regulated transport of nutrients to the retina while preserving visual function by restricting access to toxins, pathogens and immune cells. During non-infectious uveitis and its animal model EAU, leukocytes infiltrate the retina through a disrupted BRB and induce retinal damage. As the main component of the inner BRB, retinal ECs are thus a central player in the development of retinal inflammation. However, the molecular basis of BRB breakdown is only partially understood.

In this study, we first investigated the activation of total retinal cells during EAU, through comparative analysis of their transcriptomic profile in EAU versus naïve retinas. The analysis of transcriptome data reveals significant regulation (mostly *up-*regulation) of numerous genes implicated in Ag presentation in association to MHC Class II and T cell activation. This mainly reflects local engagement of the adaptive immune response, whose function in EAU development is already well-known [29]. Some of those genes have already been studied as potential therapeutic targets during EAU, e.g. Icos [30], IL17R [30] or CTLA4 [31] but numerous genes have not yet been explored in the field of uveitis. For example, the tyrosine kinase Jak3 is activated by interleukin (IL) receptors IL2R and IL17R (that comprise a common chain) and plays a capital role for immune response initiation.

However, one pitfall of this approach is that cells isolated from EAU retinas comprise both resident retinal cells and infiltrating inflammatory cells. Furthermore, very few endothelial-specific genes are brought to light by this global analysis. We thus decided to investigate specifically the activation of retinal ECs during EAU, through comparative analysis of their transcriptomic profile in EAU versus naïve retinas.

Endothelial cell isolation is challenging. Many techniques such as magnetic purification (Magnetic-activated cell sorting, MACS), freeze-fracture method or scraping of the endothelium do not yield pure endothelial populations [28]. Furthermore, those last 2 methods are not applicable to retinal microvessels due to their small size. Contamination by other cell types may be eliminated by further selection, for example by culturing ECs under adapted cell culture conditions. However, although in vitro models represent a useful tool for functional analysis of candidate proteins and high-throughput testing, cultured ECs rapidly lose their barrier properties, such as the high number of tight junction proteins and low number of transcytosis vesicles [21]. These properties can be partially restored by adding astrocytes and pericytes to EC cultures [32], but no in vitro model ideally reconstitutes all properties observed in vivo, since hemodynamic conditions and effects of circulating cells and proteins are lacking.

Although Tie2-GFP transgenic mice have proved to be very useful for effective EC isolation [33], we experienced highly restrictive low transmission rates when trying to derive the Tie2-GFP transgene into a C57BL/6 J background, that led us to explore an alternative EC isolation strategy based only on the expression of cell-surface EC markers. Isolation of ECs often relies on CD31 expression [34]. However, our IF images provide evidence for the expression of CD31 by immune cells infiltrating the vitreous (Fig. 2a). In agreement, CD31 expression was reported in diverse immune cells [35]. Although endoglin expression has also been described in myeloid precursors and macrophages [36], in our staining experiments, endoglin expression in EAU retinal cryosections remained strictly confined to retinal vessels. We thus decided to purify ECs by flow cytometry sorting of CD31 + endoglin+ double positive CD45- cells.

In our transcriptome analysis, we detected the presence of pericytes in naïve EC samples, whether sorted from Tie2-GFP or WT mice. The intimate relationship between pericytes and ECs was initially described in the 1970s, pericytes being enclosed in the endothelial basement membrane and involved in adhesive junctions with ECs, rendering individualization of each cell type particularly tricky [37]. Pericytes are exceptionally abundant at the level of barrier-type ECs, with a pericyte/EC ratio of 1:1 in the retina and 1:3 in the brain, compared to 1:10 in other microvascular beds [21]. Although the majority of the pericyte-endothelial interface is separated by a basement membrane, at some places the two cell types form focal contacts through N-cadherin and connexins, allowing them to exchange metabolites and even ribonucleic acids [38]. Such pericyte contamination of EC samples was also observed by Daneman et al. in brain ECs sorted from Tie2-GFP transgenic mice [1]. In that study, a double FACS procedure with exclusion of cells positive for the pericyte marker PDGFRß allowed isolation of pure ECs. Unfortunately, the number of ECs that can be isolated from the retina is much lower than that obtainable from the brain, and in our experimental conditions a double FACS procedure would cause excessive cell loss. In an attempt to exclude pericytes, we tested an anti-PDGFRß antibody but observed only a very weak signal, hardly distinguishable from the FMO control (Additional file 9: Fig. S9). However, since it was shown that pericytes actively take part in establishing the barrier properties of the inner BRB [21], including pericytes in the analysis of gene regulation involved in BRB breakdown bears a lot of interest. Interestingly, our RNA-Seq data indicate lower pericyte contamination in EAU ECs in comparison with naïve ECs. This is consistent with the fact that BRB breakdown has been associated with pericyte dysfunction and loss. Pericyte loss in the brain was reported to be associated with upregulation of EC transcytosis and induction of several permeability-related factors [39]. The role of pericytes is less clear in the retina, but pericyte loss occurs early in diabetic retinopathy [21]. Our data thus suggest that pericyte loss is also implicated in uveitis-related BRB breakdown.

We also observed contamination of EC samples by photoreceptor genes. The retina is mainly composed of rods (80%) and many research teams have shown retinal cell transcriptome contamination by photoreceptor genes, even with extremely draconian sorting methods relying on transgenic mouse lines selectively expressing fluorescent proteins in different retinal cell types [29, 40, 41]. In this context, interestingly, McKenzie et al. studied the modification of retinal EC gene expression in different mutant models of non-neovascular remodeling by microarray, and also picked up photoreceptor gene expression in retinal vessel fractions [42]. Contrary to pericyte contamination, photoreceptor genes were more strongly expressed by diseased ECs compared to naïve. This could reflect the fact that diseased cells are more adhesive than naïve [43].

To bypass this contamination, we explored 2 approaches: the analysis of the 120 most variant genes across DE (diseased endothelium) and NE (naïve endothelium) samples and a bioinformatics analysis to select genes based on their expression profile. We then systematically eliminated remaining photoreceptor genes. By combining these 2 approaches, we identified 82 genes significantly modulated in DE compared to NE. Among those genes, a few are already known to be implicated in uveitis, such as E and P selectins [43], CD44 [44], IL-33 [45, 46] and Lcn2 [47]. Other genes were already implicated in other inflammatory disease models but not in uveitis, such as Lrg1 [48], Ackr1 [49] and Timp1 [50]. Finally, some of those genes are known to be involved in retinal function but not during uveitis specifically, such as Clu [51], Lrg1 [8] and Fgf2 [52].

Functional analysis of the RNA-Seq data with the DAVID web-based tool allowed to identify different pathways enriched in ECs during EAU. Among those, some enriched GO terms were quite expected, such as those related to the inflammatory response or to cell adhesion processes. Among less expected enriched pathways in diseased retinal ECs, we found upregulation of molecules related to complement activation, extracellular matrix and angiogenesis.

A few studies have already reported involvement of the complement cascade in uveitis development [53–55], as well as in EAE pathogenesis [56].

Disruption of basement membrane and extracellular matrix components is required for immune cell migration towards inflamed sites. Proteolysis by matrix metalloproteinases (MMPs) is also involved in the regulation of EC barrier function, as well in other processes such as vascular growth and interaction with circulating immune cells [57]. Elevated levels of MMPs were found in the aqueous humor of uveitis patients, in correlation with the inflammatory activity [58, 59], and it was shown that specific inhibition of MMP-2 and -9 ameliorates EAU [60].

Angiogenesis is not typically associated with peak EAU. Interestingly, however, the major angiogenic factor VEGF was shown to be increased in the retina during EAU without neovascularization and involved in induction of vascular permeability, highlighting a less described implication in other processes than angiogenesis [61]. The association of sustained inflammation with angiogenesis is now well established [62] and an interplay is described in the pathogenesis of major retinal diseases. These connections between inflammation and angiogenesis are reflected by the efficacy of steroids in diabetic retinopathy [63] and of anti-VEGF in some uveitis patients [64].

Interestingly, to date, in the literature, the results of gene and protein profiling do not correlate very well [56]. Therefore, we used IF imaging on both retinal cryosections and retinal wholemounts as well as flow cytometry to validate the expression of some attractive regulated genes we identified by RNA-Seq.

Our RNA-Seq data point out an expression of serpina3n in both retinal ECs and total retina during EAU. IF images confirm that expression is strongly induced both at the vascular level and on glial cells during EAU. Serpina3n is a secreted serine protease inhibitor associated to the inflammatory response, whose expression was demonstrated in the ischemic brain and in the liver and pancreas in response to inflammatory stimuli [65]. In agreement with our data, Takamiya et al. showed that serpina3n mRNA is induced at the perivascular level, in retinal astrocytes and uveal epithelial cells during the early phase of endotoxin-induced uveitis in rats [65]. Interestingly, serpina3n was shown to have neuroprotective effects in the EAE model, through inhibition of granzyme B [66]. Genes of the serpin family were recently found to have an altered expression in different neurological disease models, with specific involvement of serpina3n in EAE [67]. Our data suggest the potential interest of exploring serpina3n role in uveitis development.

According to our RNA-Seq data, lcn2 expression is induced in both retinal ECs and total retina during EAU. Our IF images clearly show upregulation of lcn2 expression by perivascular macroglial cells during EAU. However, although retinal wholemounts show vascular lcn2 expression, on retinal cryosections it seems that lcn2 expression is not truly attributable to ECs. Lcn2 was shown to be the most upregulated early stress response gene in the eye exposed to light-induced injury [68]. In agreement with our IF data, in mouse models of retinal degeneration, Müller cells were observed to respond to photoreceptor damage by expression of lcn2 [69]. Furthermore, 2 other studies in the EAE model found astrocytes and monocytes/microglia to be the major cell types expressing lcn2 [70, 71]. ECs engage in close contacts with astrocytes and Müller cells, which participate in the formation and maintenance of the BRB. These tight relationships might explain the dragging of some astrocytes and Müller cells in the EC samples during cell sorting. In the field of uveitis, lcn2 was reported to be highly induced in the equine recurrent uveitis model [72] . As concerns humans, lcn2 serum levels are increased in patients with Behçet’s disease and psoriasis compared to controls [73] . Furthermore, lcn2 concentration in aqueous humor is increased in eyes with idiopathic acute anterior uveitis [47]. At the functional level, Nam et al. found a pathogenic role for lcn2 in EAE development [70], while Berard et al. observed increased EAE severity in lcn2 knock-out (KO) mice [71]. Taken together, these data suggest an important yet to precise role of lcn2 in retinal response to stress.

Our RNA-Seq and IF data both point to ackr1 upregulation in diseased ECs compared to naïve. Ackr1 is an atypical chemokine receptor, that binds different pro-inflammatory chemokines and regulates their activity by transcytosis across the endothelium, from the basolateral to the luminal side, where those chemokines contribute to leukocyte diapedesis [74]. Endothelial expression of ackr1 was shown in other experimental models of inflammatory diseases, such as atherosclerosis [75]. Upregulation of ackr1 was shown at the level of the BBB in EAE and MS.

Our RNA-Seq and IF data agree in showing that lrg1 is upregulated in diseased endothelium compared to naïve. Lrg1 belongs to the ‘Leucine-rich Repeat’ family, whose members are known to be involved in protein-protein interactions, signal transduction, cell adhesion and development [76]. Lrg1 expression in the retina was first described by Wang et al. in 2013 [8] . In this paper, lrg1 retinal expression is shown to be restricted almost exclusively to the vasculature and strongly upregulated in different mouse models of choroidal and retinal neovascularization, where it promotes angiogenesis [8] . Besides, lrg1 expression is upregulated during acute inflammation in mice, and it was proposed that it might even serve as a diagnostic inflammatory biomarker [77] . Lrg1 expression was shown to be associated with disease activity in different human diseases such as rheumatoid arthritis [78], ulcerative colitis [79] and Crohn’s disease [48] . Our data bring to light the potential role of lrg1 in the development of retinal inflammation.

Protein expression analysis for those 4 genes thus globally confirms vascular upregulation during EAU. However, serpina3n and lcn2 are also detected at the perivascular level, mainly on glial cells. Such perivascular expression probably exists for other genes in our list. However, as previously mentioned for pericytes, macroglial cells being part of the inner BRB, study of gene regulation at their level is also clearly relevant in the context of our work.

Unlike the previous genes for which protein validation was performed, RNA-Seq data point to downregulation of lamc3 expression in DE compared to NE. Although downregulation is not clearly observed by IF, flow cytometry data show a tendency of ECs to downregulate lamc3 expression during EAU. Lamc3 is a poorly described member of the laminin family, which are a major component of the vascular basement membrane. Lamc3 was shown to play a role in vascular branching and EC proliferation during angiogenesis, though interaction with microglial cells [80].

Our RNA-Seq data confirm the absence of MHC Class II expression in ECs, even during EAU. The only candidate gene possibly involved in Ag presentation is Cathepsin S (Ctss). However, Ctss was also reported to be implicated in EC dysfunction and in particular in microvascular complications of diabetes [81].

In our data, most genes were upregulated in DE compared to NE. Functional analysis of our list of candidate genes seems to indicate that some ‘permeability’ genes are upregulated in retinal ECs during BRB breakdown. Munji et al. performed vast transcriptome studies on isolated BBB ECs in experimental models of different BBB breakdown-associated diseases: stroke, MS, brain injury and epilepsy. With this approach, they identified a set of genes whose altered expression is shared between models, that was named the *BBB dysfunction module* [67]. Interestingly, this dysfunction module contained mainly peripheral endothelial genes, suggesting that BBB breakdown rather implies upregulation of ‘permeability’ genes than downregulation of ‘barrier’ genes. No study has investigated yet whether this might also be the case in BRB breakdown.

## Conclusions

In conclusion, our work provides the first transcriptomic study on total retinal cells and retinal ECs during EAU. Our data not only corroborate the involvement of molecules known for a pathogenic role in retinal inflammation, but further provide a list of new molecules regulated in inner BRB cells during uveitis. These molecules represent potential novel therapeutic targets for the development of biologic drugs to treat human inflammatory eye disorders.

## Supplementary information

**Additional file 1. **FVB/N mice are resistant to experimental autoimmune uveitis. Classical experimental autoimmune uveitis (EAU) was induced in FVB/N mice (*n* = 5) by immunization with a subcutaneous injection of IRBP1–20 and CFA and an intraperitoneal injection of PTX. C57BL/6 mice were immunized as controls (*n* = 14). Clinical grading was performed by examination of the fundus 21 days after disease induction. Each symbol represents the mean of the clinical scores obtained for the 2 eyes of one mouse. Horizontal bars correspond to the median for each group. *p < 0,05.

**Additional file 2.** Transmission rate of the Tie2-GFP allele with successive backcrossing generations into a C57BL/6 background. C57BL/6-Tie2-GFP mice were generated by crossing of FVB/N Tie2-GFP males with WT C57BL/6 J female mice (to generate F1) and subsequent backcrossing of genetically selected Tie2-GFP carrier males with WT C57BL/6 J females over 10 generations. Mice were genotyped by PCR on genomic DNA extracted from tail samples.

**Additional file 3. **Sorting of retinal endothelial cells with the wild type strategy. a *Flow cytometry sorting strategy illustrated on a WT retina*: retinas of C57BL/6 WT mice were carefully dissected, cut into small pieces and dissociated by incubation with Liberase DL and DNase I at 37 °C for 45 min. The single cell suspensions, excluding dead cells (DAPI+), were analyzed by flow cytometry for CD45, CD31 and endoglin detection using fluorochrome-conjugated specific antibodies. Only CD45- cells are shown. b *Flow cytometry sorting strategies illustrated on a heterozygous Tie2-GFP FVB/N-C57BL/6 retina:* a first gate was placed on CD31+ CD45- cells. Among those gated cells, 92% express both GFP and endoglin. Cells that would be sorted according to the transgenic strategy are gated in orange. Cells that would be sorted according to the WT strategy are gated in blue.

**Additional file 4.** Comparison of transcriptional profiles between replicates, biological duplicates and different samples. Data are represented as a dot plot on a logarithmic scale. a. Correlation in gene expression between 2 samples of naive retinal cells, sorted from the same pool of mice. b. Correlation in gene expression between 2 samples of naive retinal cells, sorted from different pools of mice. c. Correlation in gene expression between 1 sample of diseased retinal endothelial cells and 1 sample of naive retinal cells. NR = naïve retina, DE = diseased endothelium.

**Additional file 5.** Integration of RNAseq data into a schematic table of the 182 genes significantly regulated between diseased and naive retinal cells.

**Additional file 6.** Expression of markers used for cell sorting at the mRNA level. Data are represented as boxplots of normalized mRNA expression levels (presented as Log_2_FPKM). DE = diseased endothelial cells, NE = naïve endothelial cells.

**Additional file 7.** Table showing the 21 photoreceptor genes eliminated from the list of candidate genes. Photoreceptor genes were eliminated from the list of genes that were previously selected through the approach by expression profile (green), through the approach by variance (orange) or though both approaches (grey).

**Additional file 8.** List of the 82 candidate genes. 82 candidate genes were chosen based on the 2 selection strategies (by variance and/or by expression profile) and ranked by foldchange. Genes in grey correspond to those selected through both analyses. Genes in green were identified through the analysis by expression profile and those in orange through the analysis by variance.

**Additional file 9.** Flow cytometry analysis of PDGFRß expression by retinal cells. Retinas of C57BL/6 WT mice were carefully dissected, cut into small pieces and dissociated by incubation with Liberase DL and DNase I at 37 °C for 45 min. The single cell suspensions, excluding dead cells (DAPI+) were analyzed by flow cytometry for CD45, CD31, endoglin and PDGFRß expression using fluorochrome-conjugated specific antibodies. A fluorescence minus one (FMO) control was used for accurate gating (left).

## Data Availability

The data discussed in this publication have been deposited in NCBI’s Gene Expression Omnibus [82] and are accessible through GEO Series accession number GSE144168 (https://www.ncbi.nlm.nih.gov/geo/query/acc.cgi?acc=GSE144168).

## Abbreviations

Ackr1: atypical chemokine receptor-1 (a.k.a. Duffy antigen receptor for chemokines or DARC)
AT: adoptive transfer
BBB: blood-brain barrier
BRB: blood-retinal barrier
CD: cluster differentiation
CD31: PECAM-1, platelet endothelial cell adhesion molecule
CFA: Complete Freund’s Adjuvant
DE: diseased endothelial cells
DR: diseased retinal cells
EAE: experimental autoimmune encephalomyelitis
EAU: experimental autoimmune uveitis
EC: endothelial cell
FACS: fluorescence-activated cell sorting
FC: foldchange
FDR: false detection rate
FMO: fluorescence minus one
FPKM: fragments per kilobase of exon per million mapped reads
GFP: green fluorescent protein
IF: immunofluorescence
IL: interleukin
IRBP: interphotoreceptor retinoid-binding protein
KO: knock-out
Lamc3: Laminin Subunit Gamma 3
Lcn2: lipocalin 2
Lrg1: leucine rich alpha-2-glycoprotein 1
MACS: magnetic-activated cell sorting
MMPs: matrix metalloproteinases
MS: multiple sclerosis
NE: naïve endothelial cells
NR: naïve retinal cells
PCA: principal component analysis
PDGFRß: platelet-derived growth factor receptor ß
PFA: paraformaldehyde
PTX: pertussis toxin
RPE: retinal pigment epithelium
Serpina3n: serpin family A member 3
Tie2: Tek receptor tyrosine kinase
TIMPs: tissue inhibitors of metalloproteinases
WT: wild type
