# Correction: Retinal endothelial cell phenotypic modifications during experimental autoimmune uveitis: a transcriptomic approach

**DOI:** 10.1186/s12886-023-02836-1

**Published:** 2023-03-14

**Authors:** Deborah A. Lipski, Vincent Foucart, Rémi Dewispelaere, Laure E. Caspers, Matthieu Defrance, Catherine Bruyns, François Willermain

**Affiliations:** 1 Ophthalmology Group, IRIBHM (Institut de Recherche Interdisciplinaire en Biologie Humaine et Moléculaire), Université Libre de Bruxelles (ULB), Erasme Campus, Building C, Room C6.117, 808 Route de Lennik, 1070 Brussels, Belgium; 2 Ophthalmology Department of Erasme Hospital, Université Libre de Bruxelles (ULB), 808 Route de Lennik, 1070 Brussels, Belgium; 3 Ophthalmology Department of CHU Saint-Pierre, 322 Rue Haute, 1000 Brussels, Belgium; 4 Ophthalmology Department of CHU Brugmann, 4 Place Van Gehuchten, 1020 Brussels, Belgium; 5 Interuniversity Institute of Bioinformatics in Brussels, Université Libre de Bruxelles - Vrije Universiteit Brussel, La Plaine Campus, BC building, 6th floor, CP 263, Triomflaan, 1050 Brussels, Belgium

**Correction: BMC Ophthalmol 20, 106 (2020)**

**https://doi.org/10.1186/s12886-020-1333-5**

In the original version of this article [1], the first sentence of the Methods section incorrectly described the sequence of Interphotoreceptor retinoid-binding peptide (IRBP) 1-20 as GPTHLFQPSLVLDMAKVLLD.

The correct sequence is Ac-GPTHLFQPSLVLDMAKVLD-amide.

The PDF and HTML versions of the original article have been corrected.
