# Supplementary figures and images for: Retinal endothelial cell phenotypic modifications during experimental autoimmune uveitis: a transcriptomic approach
Source: BMC Ophthalmol. 2020 Mar 17;20:106. doi: 10.1186/s12886-020-1333-5 (PMC7076950; doi:10.1186/s12886-020-1333-5)

## Slide 1
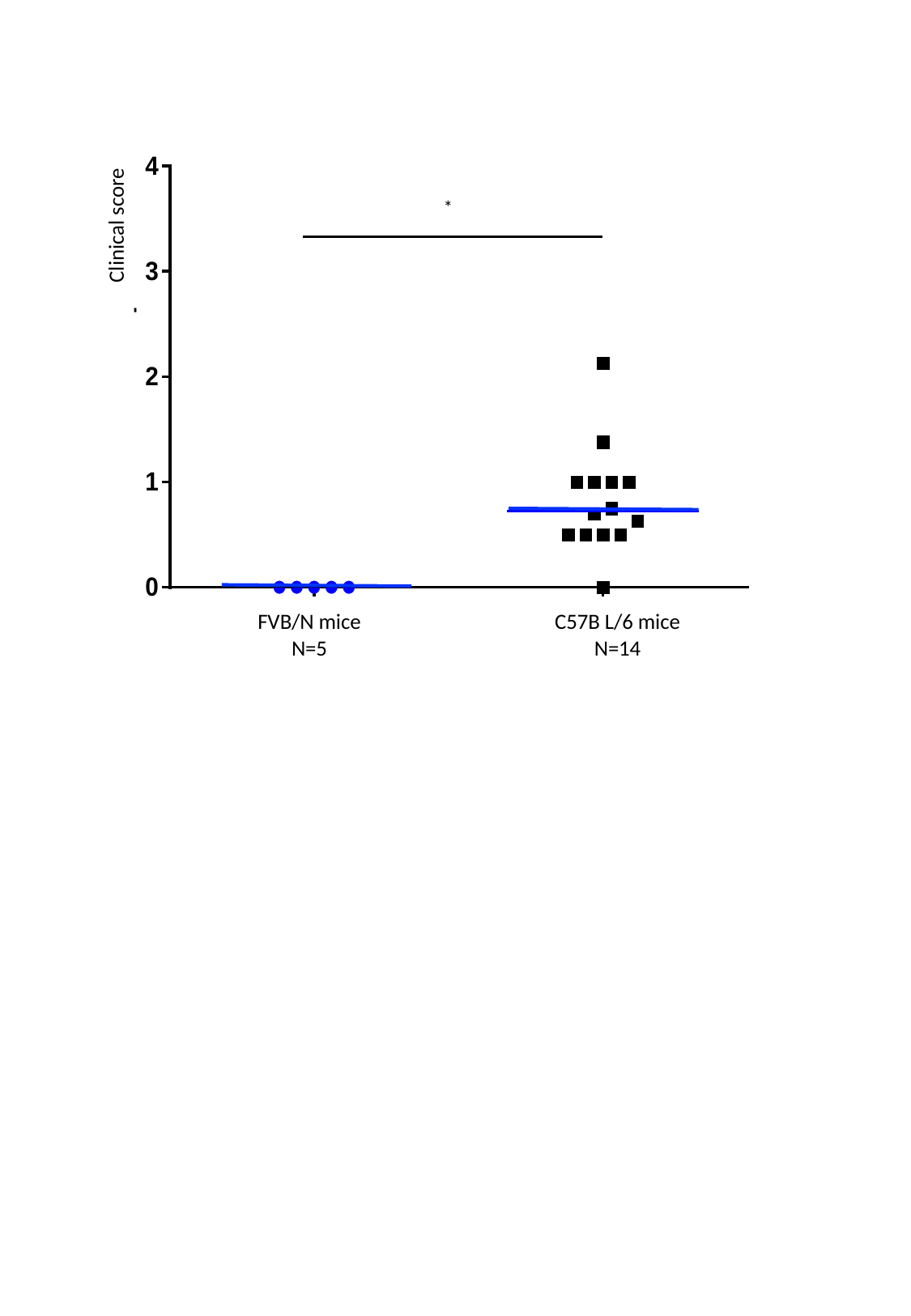

*
Clinical score
C57B L/6 mice
N=14
FVB/N mice
N=5

Supplement: Supplementary file 1 — Additional file 1. FVB/N mice are resistant to experimental autoimmune uveitis. Classical experimental autoimmune uveitis (EAU) was induced in FVB/N mice (n = 5) by immunization with a subcutaneous injection of IRBP1–20 and CFA and an intraperitoneal injection of PTX. C57BL/6 mice were immunized as controls (n = 14). Clinical grading was performed by examination of the fundus 21 days after disease induction. Each symbol represents the mean of the clinical scores obtained for the 2 eyes of one mouse. Horizontal bars correspond to the median for each group. *p < 0,05. [file 12886_2020_1333_MOESM1_ESM.pptx]

## Slide 1
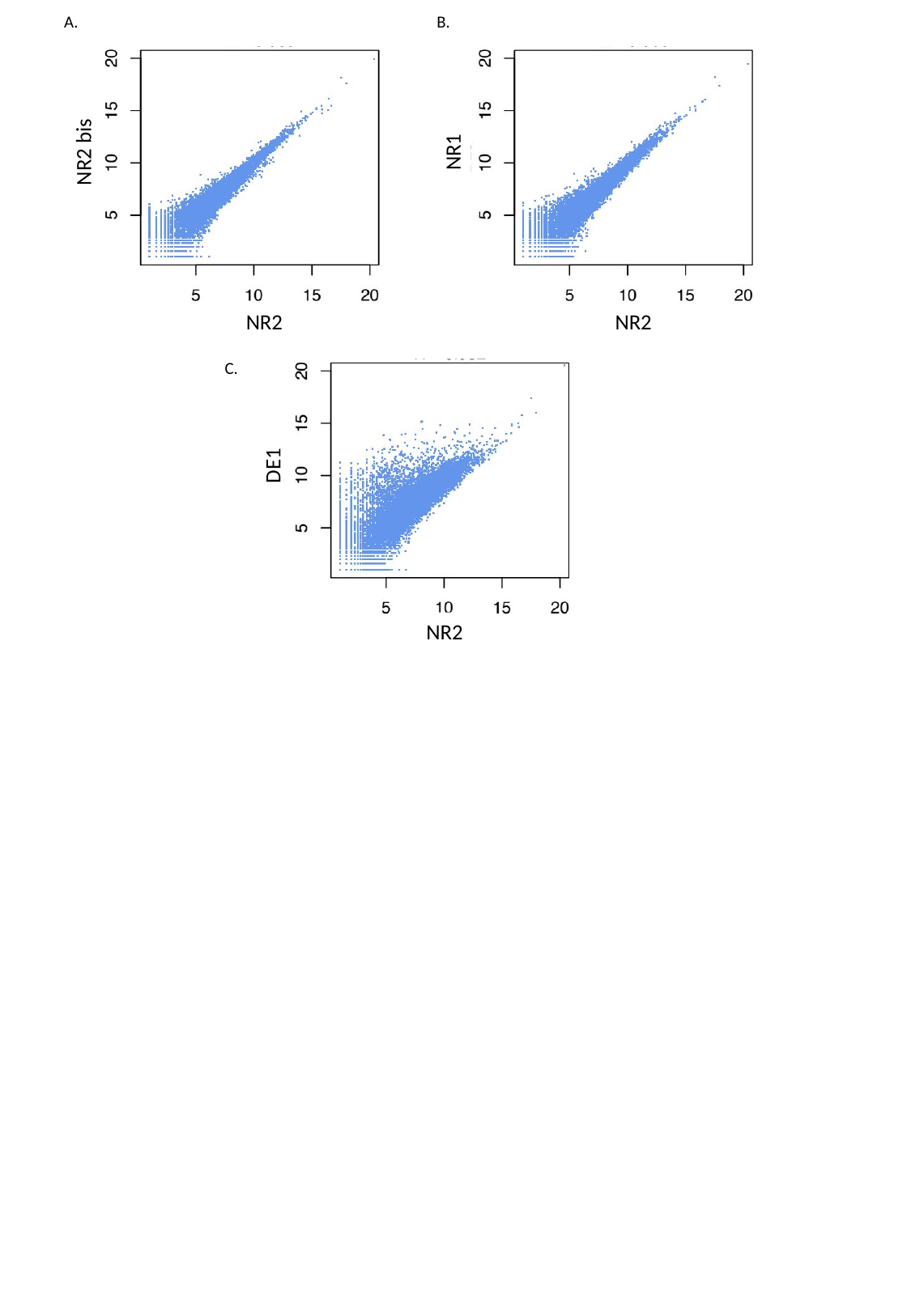

A.
B.
R = 0,989
R = 0,982
NR2 bis
NR1
NR2
NR2
R = 0,966
C.
DE1
NR2

Supplement: Supplementary file 4 — Additional file 4. Comparison of transcriptional profiles between replicates, biological duplicates and different samples. Data are represented as a dot plot on a logarithmic scale. a. Correlation in gene expression between 2 samples of naive retinal cells, sorted from the same pool of mice. b. Correlation in gene expression between 2 samples of naive retinal cells, sorted from different pools of mice. c. Correlation in gene expression between 1 sample of diseased retinal endothelial cells and 1 sample of naive retinal cells. NR = naïve retina, DE = diseased endothelium. [file 12886_2020_1333_MOESM4_ESM.pptx]

## Slide 1
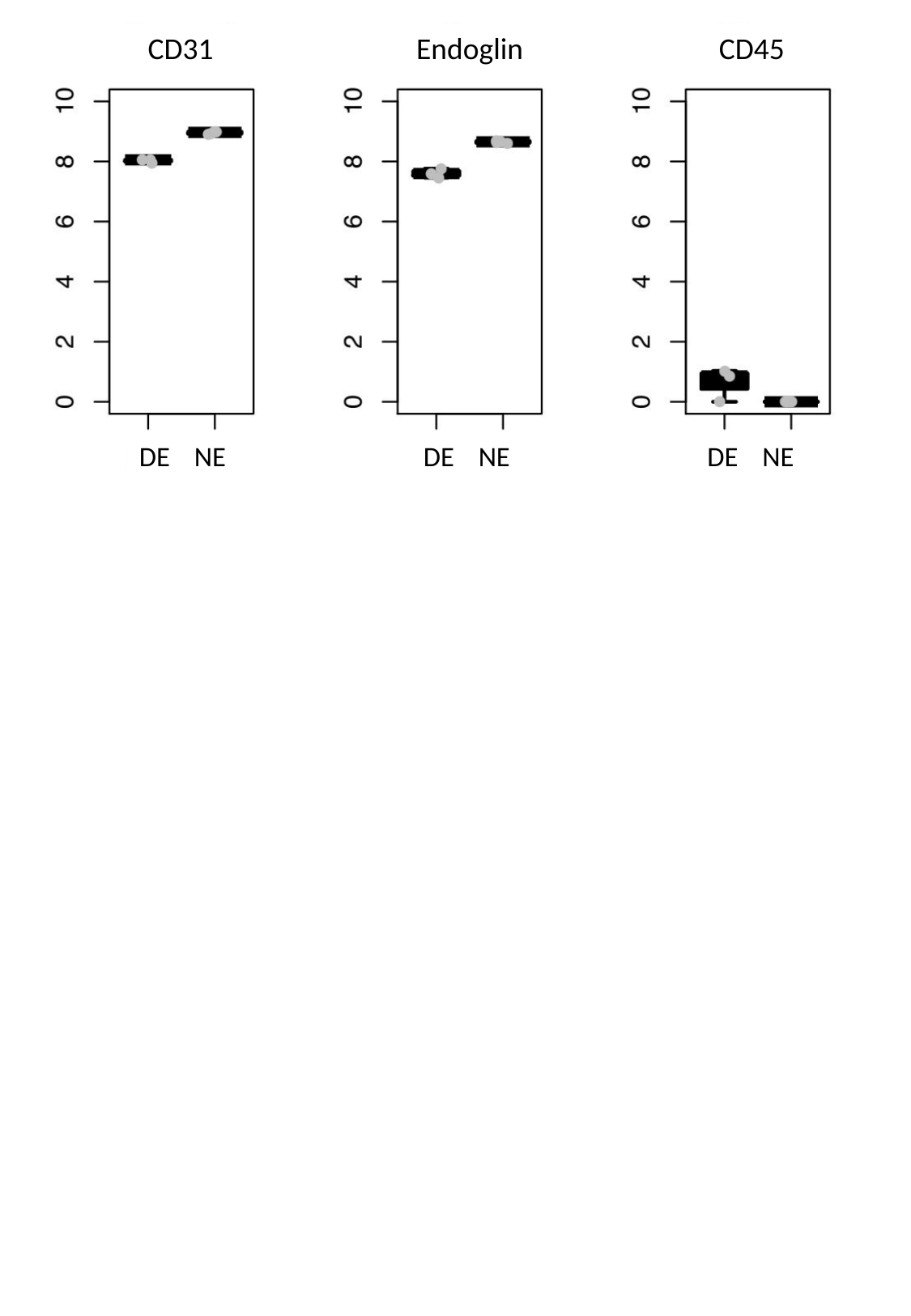

CD45
CD31
Endoglin
DE
NE
DE
NE
DE
NE

Supplement: Supplementary file 6 — Additional file 6. Expression of markers used for cell sorting at the mRNA level. Data are represented as boxplots of normalized mRNA expression levels (presented as Log2FPKM). DE = diseased endothelial cells, NE = naïve endothelial cells. [file 12886_2020_1333_MOESM6_ESM.pptx]

## Slide 1
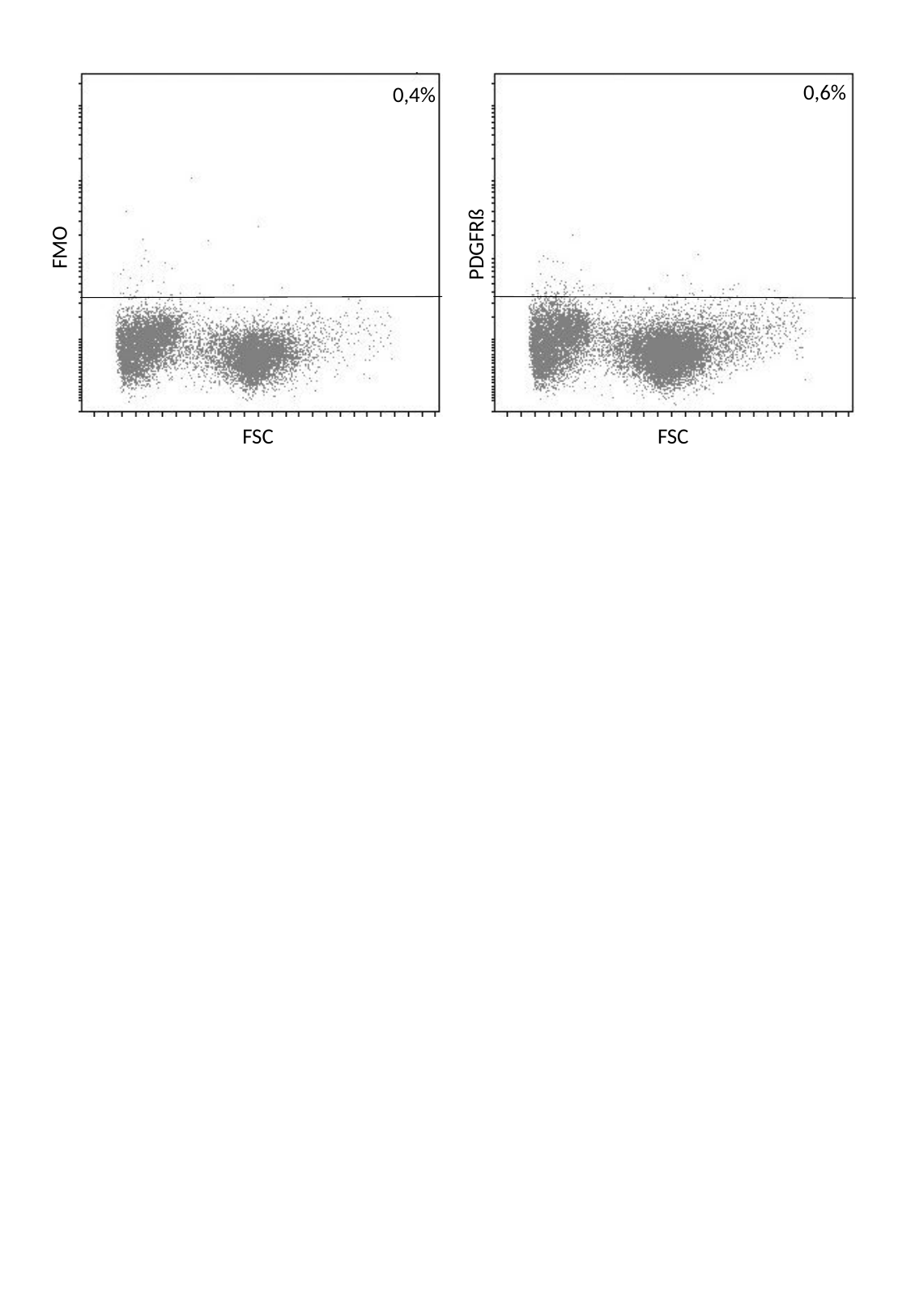

0,6%
0,4%
PDGFRß
FMO
FSC
FSC

Supplement: Supplementary file 9 — Additional file 9. Flow cytometry analysis of PDGFRß expression by retinal cells. Retinas of C57BL/6 WT mice were carefully dissected, cut into small pieces and dissociated by incubation with Liberase DL and DNase I at 37 °C for 45 min. The single cell suspensions, excluding dead cells (DAPI+) were analyzed by flow cytometry for CD45, CD31, endoglin and PDGFRß expression using fluorochrome-conjugated specific antibodies. A fluorescence minus one (FMO) control was used for accurate gating (left). [file 12886_2020_1333_MOESM9_ESM.pptx]
