# Supplementary material for: Retinal endothelial cell phenotypic modifications during experimental autoimmune uveitis: a transcriptomic approach
Source: BMC Ophthalmol. 2020 Mar 17;20:106. doi: 10.1186/s12886-020-1333-5 (PMC7076950; doi:10.1186/s12886-020-1333-5)
Supplement: Supplementary file 2 — Additional file 2. Transmission rate of the Tie2-GFP allele with successive backcrossing generations into a C57BL/6 background. C57BL/6-Tie2-GFP mice were generated by crossing of FVB/N Tie2-GFP males with WT C57BL/6 J female mice (to generate F1) and subsequent backcrossing of genetically selected Tie2-GFP carrier males with WT C57BL/6 J females over 10 generations. Mice were genotyped by PCR on genomic DNA extracted from tail samples. [file 12886_2020_1333_MOESM2_ESM.pptx]

## Slide 1
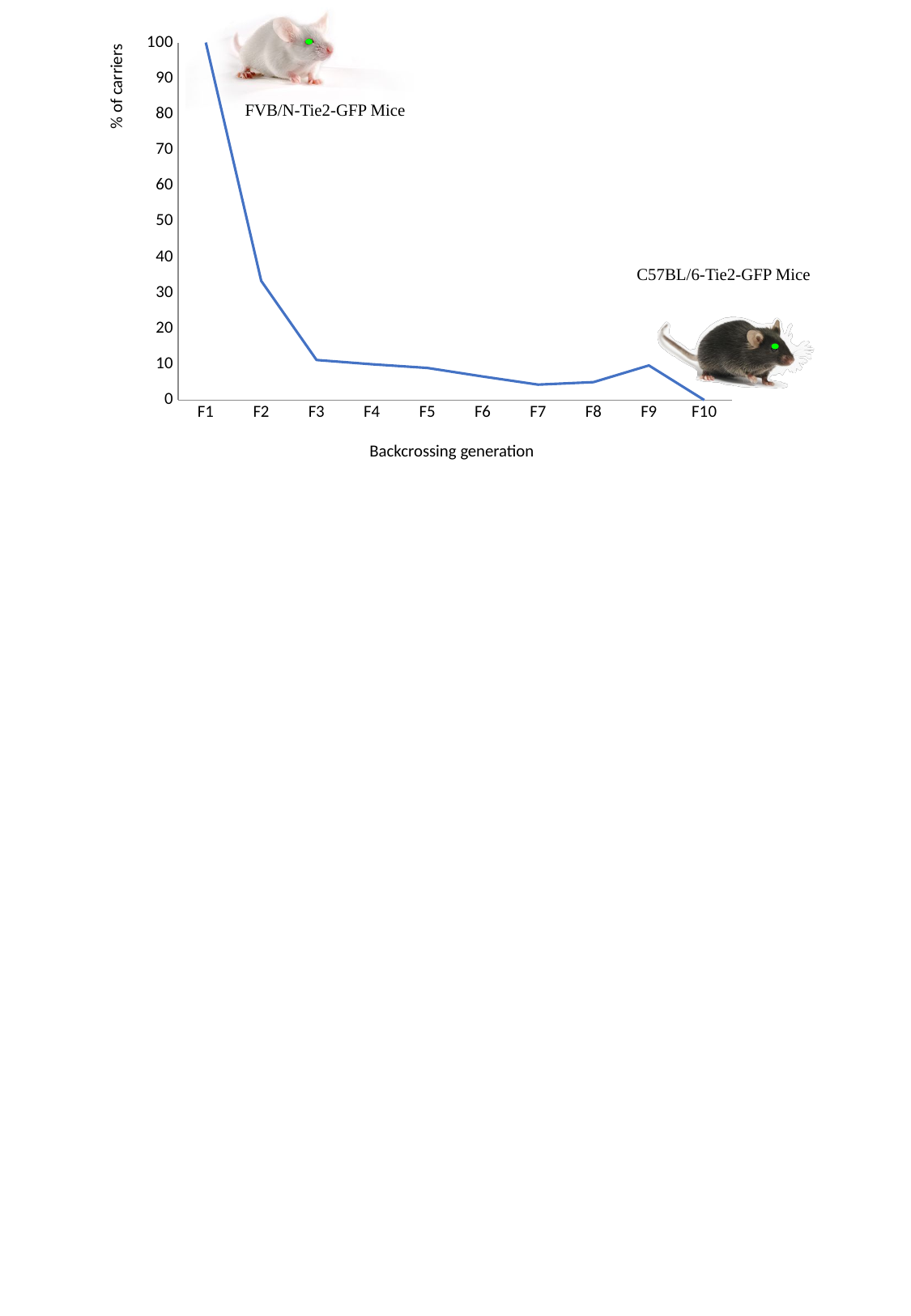

### Chart
| Category | |
|---|---|
| F1 | 100.0 |
| F2 | 33.33333333333333 |
| F3 | 11.18881118881119 |
| F4 | 10.0 |
| F5 | 8.974358974358973 |
| F6 | 6.57894736842105 |
| F7 | 4.316546762589928 |
| F8 | 5.0 |
| F9 | 9.67741935483871 |
| F10 | 0.0 |
FVB/N-Tie2-GFP Mice
% of carriers
C57BL/6-Tie2-GFP Mice
Backcrossing generation
