# Supplementary material for: Retinal endothelial cell phenotypic modifications during experimental autoimmune uveitis: a transcriptomic approach
Source: BMC Ophthalmol. 2020 Mar 17;20:106. doi: 10.1186/s12886-020-1333-5 (PMC7076950; doi:10.1186/s12886-020-1333-5)
Supplement: Supplementary file 3 — Additional file 3. Sorting of retinal endothelial cells with the wild type strategy. a Flow cytometry sorting strategy illustrated on a WT retina: retinas of C57BL/6 WT mice were carefully dissected, cut into small pieces and dissociated by incubation with Liberase DL and DNase I at 37 °C for 45 min. The single cell suspensions, excluding dead cells (DAPI+), were analyzed by flow cytometry for CD45, CD31 and endoglin detection using fluorochrome-conjugated specific antibodies. Only CD45- cells are shown. b Flow cytometry sorting strategies illustrated on a heterozygous Tie2-GFP FVB/N-C57BL/6 retina: a first gate was placed on CD31+ CD45- cells. Among those gated cells, 92% express both GFP and endoglin. Cells that would be sorted according to the transgenic strategy are gated in orange. Cells that would be sorted according to the WT strategy are gated in blue. [file 12886_2020_1333_MOESM3_ESM.pptx]

## Slide 1
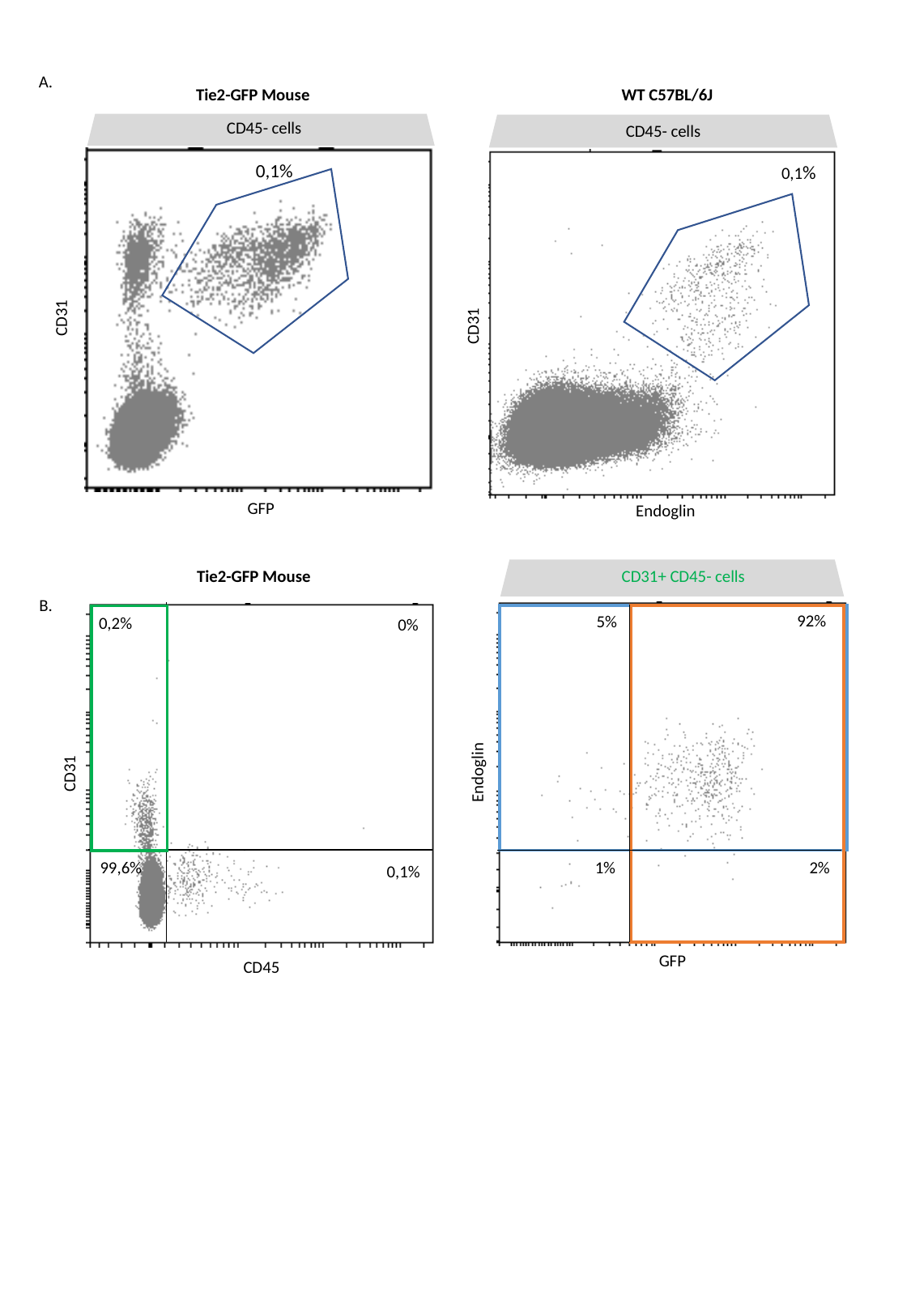

A.
Tie2-GFP Mouse
WT C57BL/6J
CD45- cells
0,1%
CD31
Endoglin
B.
CD45- cells
0,1%
CD31
GFP
CD31+ CD45- cells
92%
5%
Endoglin
1%
2%
GFP
Tie2-GFP Mouse
0,2%
0%
CD31
99,6%
0,1%
CD45
