# Supplementary material for: Retinal endothelial cell phenotypic modifications during experimental autoimmune uveitis: a transcriptomic approach
Source: BMC Ophthalmol. 2020 Mar 17;20:106. doi: 10.1186/s12886-020-1333-5 (PMC7076950; doi:10.1186/s12886-020-1333-5)
Supplement: Supplementary file 5 — Additional file 5. Integration of RNAseq data into a schematic table of the 182 genes significantly regulated between diseased and naive retinal cells. [file 12886_2020_1333_MOESM5_ESM.pptx]

## Slide 1
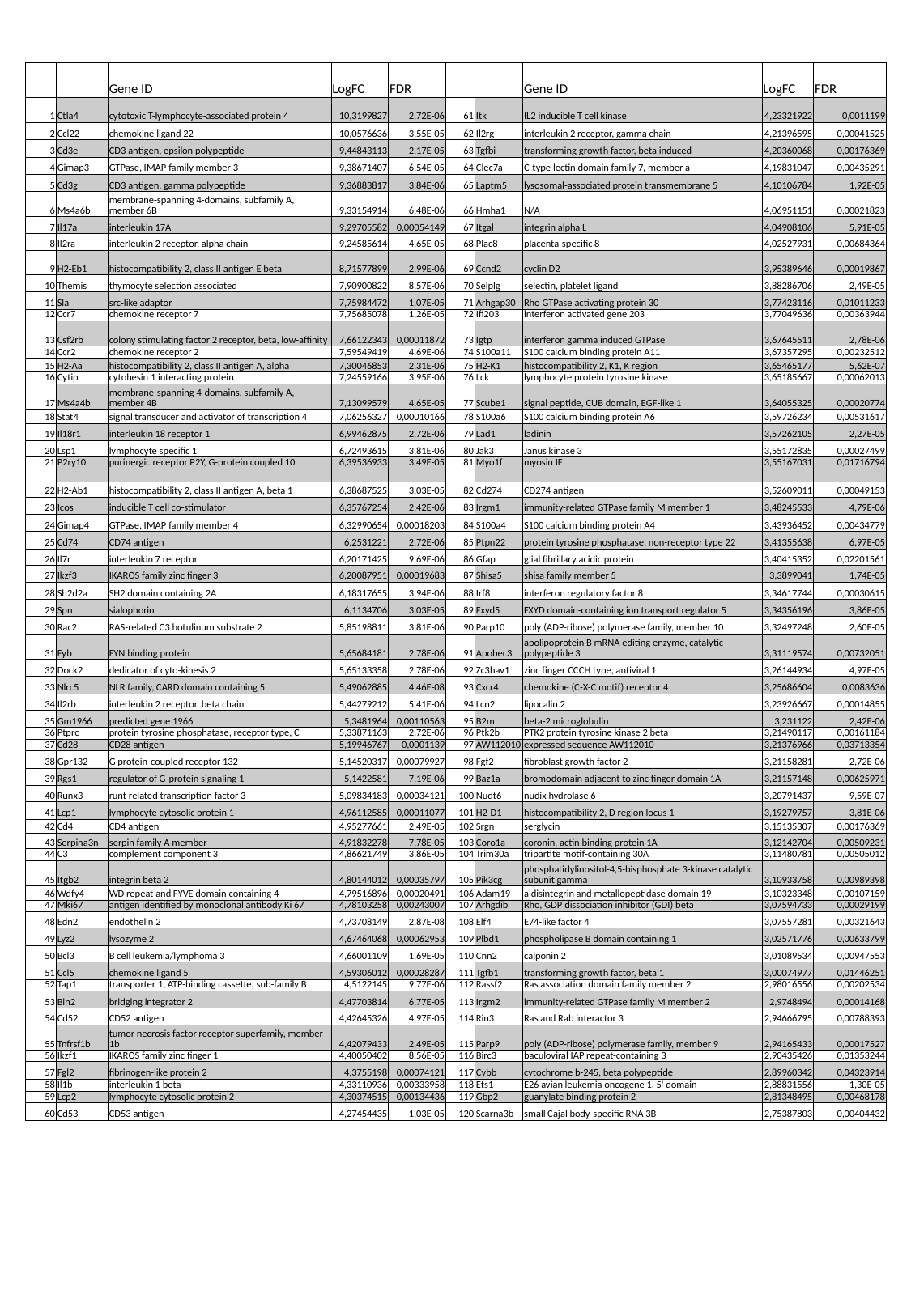

| | | Gene ID | LogFC | FDR | | | Gene ID | LogFC | FDR |
| --- | --- | --- | --- | --- | --- | --- | --- | --- | --- |
| 1 | Ctla4 | cytotoxic T-lymphocyte-associated protein 4 | 10,3199827 | 2,72E-06 | 61 | Itk | IL2 inducible T cell kinase | 4,23321922 | 0,0011199 |
| 2 | Ccl22 | chemokine ligand 22 | 10,0576636 | 3,55E-05 | 62 | Il2rg | interleukin 2 receptor, gamma chain | 4,21396595 | 0,00041525 |
| 3 | Cd3e | CD3 antigen, epsilon polypeptide | 9,44843113 | 2,17E-05 | 63 | Tgfbi | transforming growth factor, beta induced | 4,20360068 | 0,00176369 |
| 4 | Gimap3 | GTPase, IMAP family member 3 | 9,38671407 | 6,54E-05 | 64 | Clec7a | C-type lectin domain family 7, member a | 4,19831047 | 0,00435291 |
| 5 | Cd3g | CD3 antigen, gamma polypeptide | 9,36883817 | 3,84E-06 | 65 | Laptm5 | lysosomal-associated protein transmembrane 5 | 4,10106784 | 1,92E-05 |
| 6 | Ms4a6b | membrane-spanning 4-domains, subfamily A, member 6B | 9,33154914 | 6,48E-06 | 66 | Hmha1 | N/A | 4,06951151 | 0,00021823 |
| 7 | Il17a | interleukin 17A | 9,29705582 | 0,00054149 | 67 | Itgal | integrin alpha L | 4,04908106 | 5,91E-05 |
| 8 | Il2ra | interleukin 2 receptor, alpha chain | 9,24585614 | 4,65E-05 | 68 | Plac8 | placenta-specific 8 | 4,02527931 | 0,00684364 |
| 9 | H2-Eb1 | histocompatibility 2, class II antigen E beta | 8,71577899 | 2,99E-06 | 69 | Ccnd2 | cyclin D2 | 3,95389646 | 0,00019867 |
| 10 | Themis | thymocyte selection associated | 7,90900822 | 8,57E-06 | 70 | Selplg | selectin, platelet ligand | 3,88286706 | 2,49E-05 |
| 11 | Sla | src-like adaptor | 7,75984472 | 1,07E-05 | 71 | Arhgap30 | Rho GTPase activating protein 30 | 3,77423116 | 0,01011233 |
| 12 | Ccr7 | chemokine receptor 7 | 7,75685078 | 1,26E-05 | 72 | Ifi203 | interferon activated gene 203 | 3,77049636 | 0,00363944 |
| 13 | Csf2rb | colony stimulating factor 2 receptor, beta, low-affinity | 7,66122343 | 0,00011872 | 73 | Igtp | interferon gamma induced GTPase | 3,67645511 | 2,78E-06 |
| 14 | Ccr2 | chemokine receptor 2 | 7,59549419 | 4,69E-06 | 74 | S100a11 | S100 calcium binding protein A11 | 3,67357295 | 0,00232512 |
| 15 | H2-Aa | histocompatibility 2, class II antigen A, alpha | 7,30046853 | 2,31E-06 | 75 | H2-K1 | histocompatibility 2, K1, K region | 3,65465177 | 5,62E-07 |
| 16 | Cytip | cytohesin 1 interacting protein | 7,24559166 | 3,95E-06 | 76 | Lck | lymphocyte protein tyrosine kinase | 3,65185667 | 0,00062013 |
| 17 | Ms4a4b | membrane-spanning 4-domains, subfamily A, member 4B | 7,13099579 | 4,65E-05 | 77 | Scube1 | signal peptide, CUB domain, EGF-like 1 | 3,64055325 | 0,00020774 |
| 18 | Stat4 | signal transducer and activator of transcription 4 | 7,06256327 | 0,00010166 | 78 | S100a6 | S100 calcium binding protein A6 | 3,59726234 | 0,00531617 |
| 19 | Il18r1 | interleukin 18 receptor 1 | 6,99462875 | 2,72E-06 | 79 | Lad1 | ladinin | 3,57262105 | 2,27E-05 |
| 20 | Lsp1 | lymphocyte specific 1 | 6,72493615 | 3,81E-06 | 80 | Jak3 | Janus kinase 3 | 3,55172835 | 0,00027499 |
| 21 | P2ry10 | purinergic receptor P2Y, G-protein coupled 10 | 6,39536933 | 3,49E-05 | 81 | Myo1f | myosin IF | 3,55167031 | 0,01716794 |
| 22 | H2-Ab1 | histocompatibility 2, class II antigen A, beta 1 | 6,38687525 | 3,03E-05 | 82 | Cd274 | CD274 antigen | 3,52609011 | 0,00049153 |
| 23 | Icos | inducible T cell co-stimulator | 6,35767254 | 2,42E-06 | 83 | Irgm1 | immunity-related GTPase family M member 1 | 3,48245533 | 4,79E-06 |
| 24 | Gimap4 | GTPase, IMAP family member 4 | 6,32990654 | 0,00018203 | 84 | S100a4 | S100 calcium binding protein A4 | 3,43936452 | 0,00434779 |
| 25 | Cd74 | CD74 antigen | 6,2531221 | 2,72E-06 | 85 | Ptpn22 | protein tyrosine phosphatase, non-receptor type 22 | 3,41355638 | 6,97E-05 |
| 26 | Il7r | interleukin 7 receptor | 6,20171425 | 9,69E-06 | 86 | Gfap | glial fibrillary acidic protein | 3,40415352 | 0,02201561 |
| 27 | Ikzf3 | IKAROS family zinc finger 3 | 6,20087951 | 0,00019683 | 87 | Shisa5 | shisa family member 5 | 3,3899041 | 1,74E-05 |
| 28 | Sh2d2a | SH2 domain containing 2A | 6,18317655 | 3,94E-06 | 88 | Irf8 | interferon regulatory factor 8 | 3,34617744 | 0,00030615 |
| 29 | Spn | sialophorin | 6,1134706 | 3,03E-05 | 89 | Fxyd5 | FXYD domain-containing ion transport regulator 5 | 3,34356196 | 3,86E-05 |
| 30 | Rac2 | RAS-related C3 botulinum substrate 2 | 5,85198811 | 3,81E-06 | 90 | Parp10 | poly (ADP-ribose) polymerase family, member 10 | 3,32497248 | 2,60E-05 |
| 31 | Fyb | FYN binding protein | 5,65684181 | 2,78E-06 | 91 | Apobec3 | apolipoprotein B mRNA editing enzyme, catalytic polypeptide 3 | 3,31119574 | 0,00732051 |
| 32 | Dock2 | dedicator of cyto-kinesis 2 | 5,65133358 | 2,78E-06 | 92 | Zc3hav1 | zinc finger CCCH type, antiviral 1 | 3,26144934 | 4,97E-05 |
| 33 | Nlrc5 | NLR family, CARD domain containing 5 | 5,49062885 | 4,46E-08 | 93 | Cxcr4 | chemokine (C-X-C motif) receptor 4 | 3,25686604 | 0,0083636 |
| 34 | Il2rb | interleukin 2 receptor, beta chain | 5,44279212 | 5,41E-06 | 94 | Lcn2 | lipocalin 2 | 3,23926667 | 0,00014855 |
| 35 | Gm1966 | predicted gene 1966 | 5,3481964 | 0,00110563 | 95 | B2m | beta-2 microglobulin | 3,231122 | 2,42E-06 |
| 36 | Ptprc | protein tyrosine phosphatase, receptor type, C | 5,33871163 | 2,72E-06 | 96 | Ptk2b | PTK2 protein tyrosine kinase 2 beta | 3,21490117 | 0,00161184 |
| 37 | Cd28 | CD28 antigen | 5,19946767 | 0,0001139 | 97 | AW112010 | expressed sequence AW112010 | 3,21376966 | 0,03713354 |
| 38 | Gpr132 | G protein-coupled receptor 132 | 5,14520317 | 0,00079927 | 98 | Fgf2 | fibroblast growth factor 2 | 3,21158281 | 2,72E-06 |
| 39 | Rgs1 | regulator of G-protein signaling 1 | 5,1422581 | 7,19E-06 | 99 | Baz1a | bromodomain adjacent to zinc finger domain 1A | 3,21157148 | 0,00625971 |
| 40 | Runx3 | runt related transcription factor 3 | 5,09834183 | 0,00034121 | 100 | Nudt6 | nudix hydrolase 6 | 3,20791437 | 9,59E-07 |
| 41 | Lcp1 | lymphocyte cytosolic protein 1 | 4,96112585 | 0,00011077 | 101 | H2-D1 | histocompatibility 2, D region locus 1 | 3,19279757 | 3,81E-06 |
| 42 | Cd4 | CD4 antigen | 4,95277661 | 2,49E-05 | 102 | Srgn | serglycin | 3,15135307 | 0,00176369 |
| 43 | Serpina3n | serpin family A member | 4,91832278 | 7,78E-05 | 103 | Coro1a | coronin, actin binding protein 1A | 3,12142704 | 0,00509231 |
| 44 | C3 | complement component 3 | 4,86621749 | 3,86E-05 | 104 | Trim30a | tripartite motif-containing 30A | 3,11480781 | 0,00505012 |
| 45 | Itgb2 | integrin beta 2 | 4,80144012 | 0,00035797 | 105 | Pik3cg | phosphatidylinositol-4,5-bisphosphate 3-kinase catalytic subunit gamma | 3,10933758 | 0,00989398 |
| 46 | Wdfy4 | WD repeat and FYVE domain containing 4 | 4,79516896 | 0,00020491 | 106 | Adam19 | a disintegrin and metallopeptidase domain 19 | 3,10323348 | 0,00107159 |
| 47 | Mki67 | antigen identified by monoclonal antibody Ki 67 | 4,78103258 | 0,00243007 | 107 | Arhgdib | Rho, GDP dissociation inhibitor (GDI) beta | 3,07594733 | 0,00029199 |
| 48 | Edn2 | endothelin 2 | 4,73708149 | 2,87E-08 | 108 | Elf4 | E74-like factor 4 | 3,07557281 | 0,00321643 |
| 49 | Lyz2 | lysozyme 2 | 4,67464068 | 0,00062953 | 109 | Plbd1 | phospholipase B domain containing 1 | 3,02571776 | 0,00633799 |
| 50 | Bcl3 | B cell leukemia/lymphoma 3 | 4,66001109 | 1,69E-05 | 110 | Cnn2 | calponin 2 | 3,01089534 | 0,00947553 |
| 51 | Ccl5 | chemokine ligand 5 | 4,59306012 | 0,00028287 | 111 | Tgfb1 | transforming growth factor, beta 1 | 3,00074977 | 0,01446251 |
| 52 | Tap1 | transporter 1, ATP-binding cassette, sub-family B | 4,5122145 | 9,77E-06 | 112 | Rassf2 | Ras association domain family member 2 | 2,98016556 | 0,00202534 |
| 53 | Bin2 | bridging integrator 2 | 4,47703814 | 6,77E-05 | 113 | Irgm2 | immunity-related GTPase family M member 2 | 2,9748494 | 0,00014168 |
| 54 | Cd52 | CD52 antigen | 4,42645326 | 4,97E-05 | 114 | Rin3 | Ras and Rab interactor 3 | 2,94666795 | 0,00788393 |
| 55 | Tnfrsf1b | tumor necrosis factor receptor superfamily, member 1b | 4,42079433 | 2,49E-05 | 115 | Parp9 | poly (ADP-ribose) polymerase family, member 9 | 2,94165433 | 0,00017527 |
| 56 | Ikzf1 | IKAROS family zinc finger 1 | 4,40050402 | 8,56E-05 | 116 | Birc3 | baculoviral IAP repeat-containing 3 | 2,90435426 | 0,01353244 |
| 57 | Fgl2 | fibrinogen-like protein 2 | 4,3755198 | 0,00074121 | 117 | Cybb | cytochrome b-245, beta polypeptide | 2,89960342 | 0,04323914 |
| 58 | Il1b | interleukin 1 beta | 4,33110936 | 0,00333958 | 118 | Ets1 | E26 avian leukemia oncogene 1, 5' domain | 2,88831556 | 1,30E-05 |
| 59 | Lcp2 | lymphocyte cytosolic protein 2 | 4,30374515 | 0,00134436 | 119 | Gbp2 | guanylate binding protein 2 | 2,81348495 | 0,00468178 |
| 60 | Cd53 | CD53 antigen | 4,27454435 | 1,03E-05 | 120 | Scarna3b | small Cajal body-specific RNA 3B | 2,75387803 | 0,00404432 |

## Slide 2
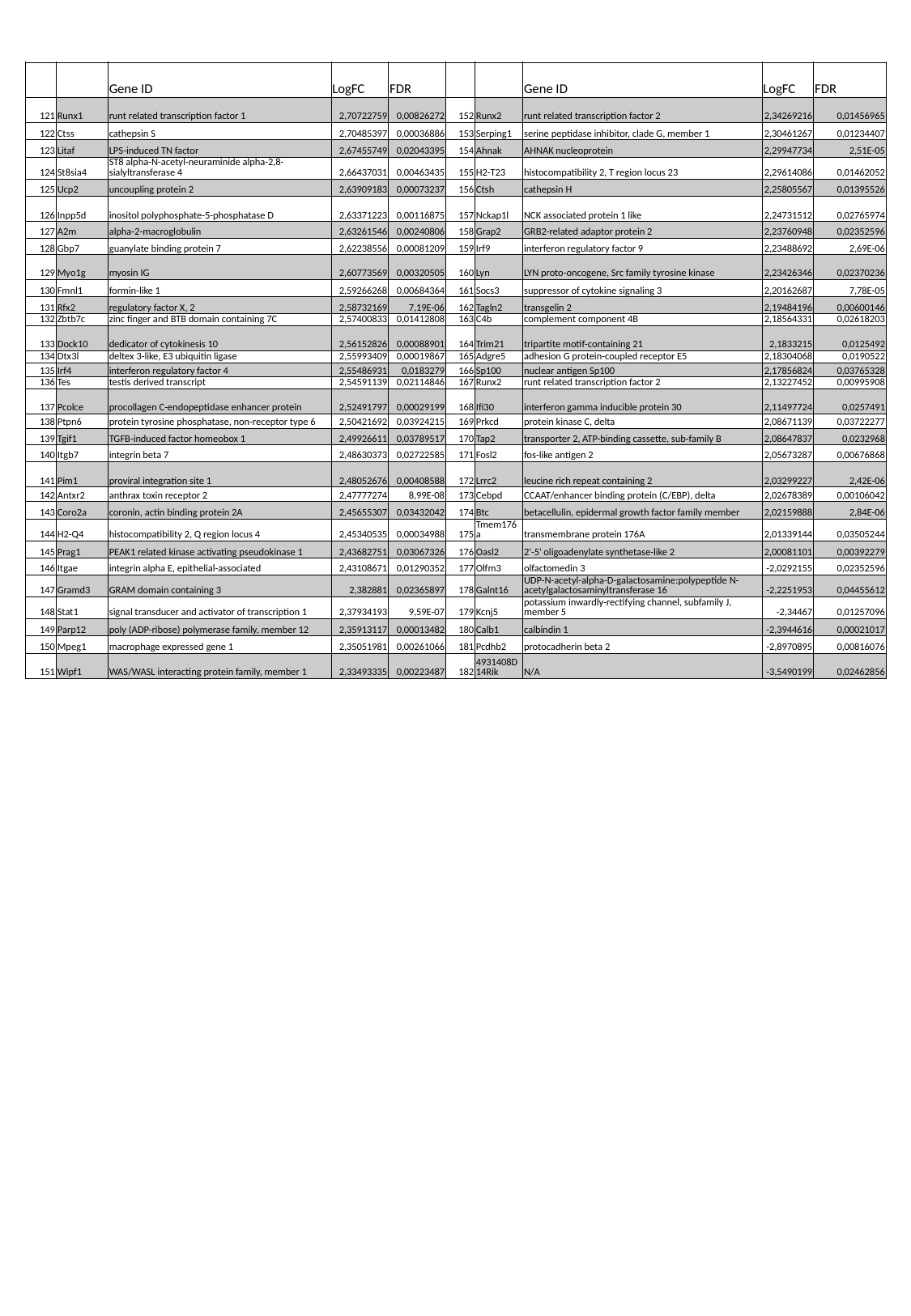

| | | Gene ID | LogFC | FDR | | | Gene ID | LogFC | FDR |
| --- | --- | --- | --- | --- | --- | --- | --- | --- | --- |
| 121 | Runx1 | runt related transcription factor 1 | 2,70722759 | 0,00826272 | 152 | Runx2 | runt related transcription factor 2 | 2,34269216 | 0,01456965 |
| 122 | Ctss | cathepsin S | 2,70485397 | 0,00036886 | 153 | Serping1 | serine peptidase inhibitor, clade G, member 1 | 2,30461267 | 0,01234407 |
| 123 | Litaf | LPS-induced TN factor | 2,67455749 | 0,02043395 | 154 | Ahnak | AHNAK nucleoprotein | 2,29947734 | 2,51E-05 |
| 124 | St8sia4 | ST8 alpha-N-acetyl-neuraminide alpha-2,8-sialyltransferase 4 | 2,66437031 | 0,00463435 | 155 | H2-T23 | histocompatibility 2, T region locus 23 | 2,29614086 | 0,01462052 |
| 125 | Ucp2 | uncoupling protein 2 | 2,63909183 | 0,00073237 | 156 | Ctsh | cathepsin H | 2,25805567 | 0,01395526 |
| 126 | Inpp5d | inositol polyphosphate-5-phosphatase D | 2,63371223 | 0,00116875 | 157 | Nckap1l | NCK associated protein 1 like | 2,24731512 | 0,02765974 |
| 127 | A2m | alpha-2-macroglobulin | 2,63261546 | 0,00240806 | 158 | Grap2 | GRB2-related adaptor protein 2 | 2,23760948 | 0,02352596 |
| 128 | Gbp7 | guanylate binding protein 7 | 2,62238556 | 0,00081209 | 159 | Irf9 | interferon regulatory factor 9 | 2,23488692 | 2,69E-06 |
| 129 | Myo1g | myosin IG | 2,60773569 | 0,00320505 | 160 | Lyn | LYN proto-oncogene, Src family tyrosine kinase | 2,23426346 | 0,02370236 |
| 130 | Fmnl1 | formin-like 1 | 2,59266268 | 0,00684364 | 161 | Socs3 | suppressor of cytokine signaling 3 | 2,20162687 | 7,78E-05 |
| 131 | Rfx2 | regulatory factor X, 2 | 2,58732169 | 7,19E-06 | 162 | Tagln2 | transgelin 2 | 2,19484196 | 0,00600146 |
| 132 | Zbtb7c | zinc finger and BTB domain containing 7C | 2,57400833 | 0,01412808 | 163 | C4b | complement component 4B | 2,18564331 | 0,02618203 |
| 133 | Dock10 | dedicator of cytokinesis 10 | 2,56152826 | 0,00088901 | 164 | Trim21 | tripartite motif-containing 21 | 2,1833215 | 0,0125492 |
| 134 | Dtx3l | deltex 3-like, E3 ubiquitin ligase | 2,55993409 | 0,00019867 | 165 | Adgre5 | adhesion G protein-coupled receptor E5 | 2,18304068 | 0,0190522 |
| 135 | Irf4 | interferon regulatory factor 4 | 2,55486931 | 0,0183279 | 166 | Sp100 | nuclear antigen Sp100 | 2,17856824 | 0,03765328 |
| 136 | Tes | testis derived transcript | 2,54591139 | 0,02114846 | 167 | Runx2 | runt related transcription factor 2 | 2,13227452 | 0,00995908 |
| 137 | Pcolce | procollagen C-endopeptidase enhancer protein | 2,52491797 | 0,00029199 | 168 | Ifi30 | interferon gamma inducible protein 30 | 2,11497724 | 0,0257491 |
| 138 | Ptpn6 | protein tyrosine phosphatase, non-receptor type 6 | 2,50421692 | 0,03924215 | 169 | Prkcd | protein kinase C, delta | 2,08671139 | 0,03722277 |
| 139 | Tgif1 | TGFB-induced factor homeobox 1 | 2,49926611 | 0,03789517 | 170 | Tap2 | transporter 2, ATP-binding cassette, sub-family B | 2,08647837 | 0,0232968 |
| 140 | Itgb7 | integrin beta 7 | 2,48630373 | 0,02722585 | 171 | Fosl2 | fos-like antigen 2 | 2,05673287 | 0,00676868 |
| 141 | Pim1 | proviral integration site 1 | 2,48052676 | 0,00408588 | 172 | Lrrc2 | leucine rich repeat containing 2 | 2,03299227 | 2,42E-06 |
| 142 | Antxr2 | anthrax toxin receptor 2 | 2,47777274 | 8,99E-08 | 173 | Cebpd | CCAAT/enhancer binding protein (C/EBP), delta | 2,02678389 | 0,00106042 |
| 143 | Coro2a | coronin, actin binding protein 2A | 2,45655307 | 0,03432042 | 174 | Btc | betacellulin, epidermal growth factor family member | 2,02159888 | 2,84E-06 |
| 144 | H2-Q4 | histocompatibility 2, Q region locus 4 | 2,45340535 | 0,00034988 | 175 | Tmem176a | transmembrane protein 176A | 2,01339144 | 0,03505244 |
| 145 | Prag1 | PEAK1 related kinase activating pseudokinase 1 | 2,43682751 | 0,03067326 | 176 | Oasl2 | 2'-5' oligoadenylate synthetase-like 2 | 2,00081101 | 0,00392279 |
| 146 | Itgae | integrin alpha E, epithelial-associated | 2,43108671 | 0,01290352 | 177 | Olfm3 | olfactomedin 3 | -2,0292155 | 0,02352596 |
| 147 | Gramd3 | GRAM domain containing 3 | 2,382881 | 0,02365897 | 178 | Galnt16 | UDP-N-acetyl-alpha-D-galactosamine:polypeptide N-acetylgalactosaminyltransferase 16 | -2,2251953 | 0,04455612 |
| 148 | Stat1 | signal transducer and activator of transcription 1 | 2,37934193 | 9,59E-07 | 179 | Kcnj5 | potassium inwardly-rectifying channel, subfamily J, member 5 | -2,34467 | 0,01257096 |
| 149 | Parp12 | poly (ADP-ribose) polymerase family, member 12 | 2,35913117 | 0,00013482 | 180 | Calb1 | calbindin 1 | -2,3944616 | 0,00021017 |
| 150 | Mpeg1 | macrophage expressed gene 1 | 2,35051981 | 0,00261066 | 181 | Pcdhb2 | protocadherin beta 2 | -2,8970895 | 0,00816076 |
| 151 | Wipf1 | WAS/WASL interacting protein family, member 1 | 2,33493335 | 0,00223487 | 182 | 4931408D14Rik | N/A | -3,5490199 | 0,02462856 |
