# Supplementary material for: Retinal endothelial cell phenotypic modifications during experimental autoimmune uveitis: a transcriptomic approach
Source: BMC Ophthalmol. 2020 Mar 17;20:106. doi: 10.1186/s12886-020-1333-5 (PMC7076950; doi:10.1186/s12886-020-1333-5)
Supplement: Supplementary file 7 — Additional file 7. Table showing the 21 photoreceptor genes eliminated from the list of candidate genes. Photoreceptor genes were eliminated from the list of genes that were previously selected through the approach by expression profile (green), through the approach by variance (orange) or though both approaches (grey). [file 12886_2020_1333_MOESM7_ESM.pptx]

## Slide 1
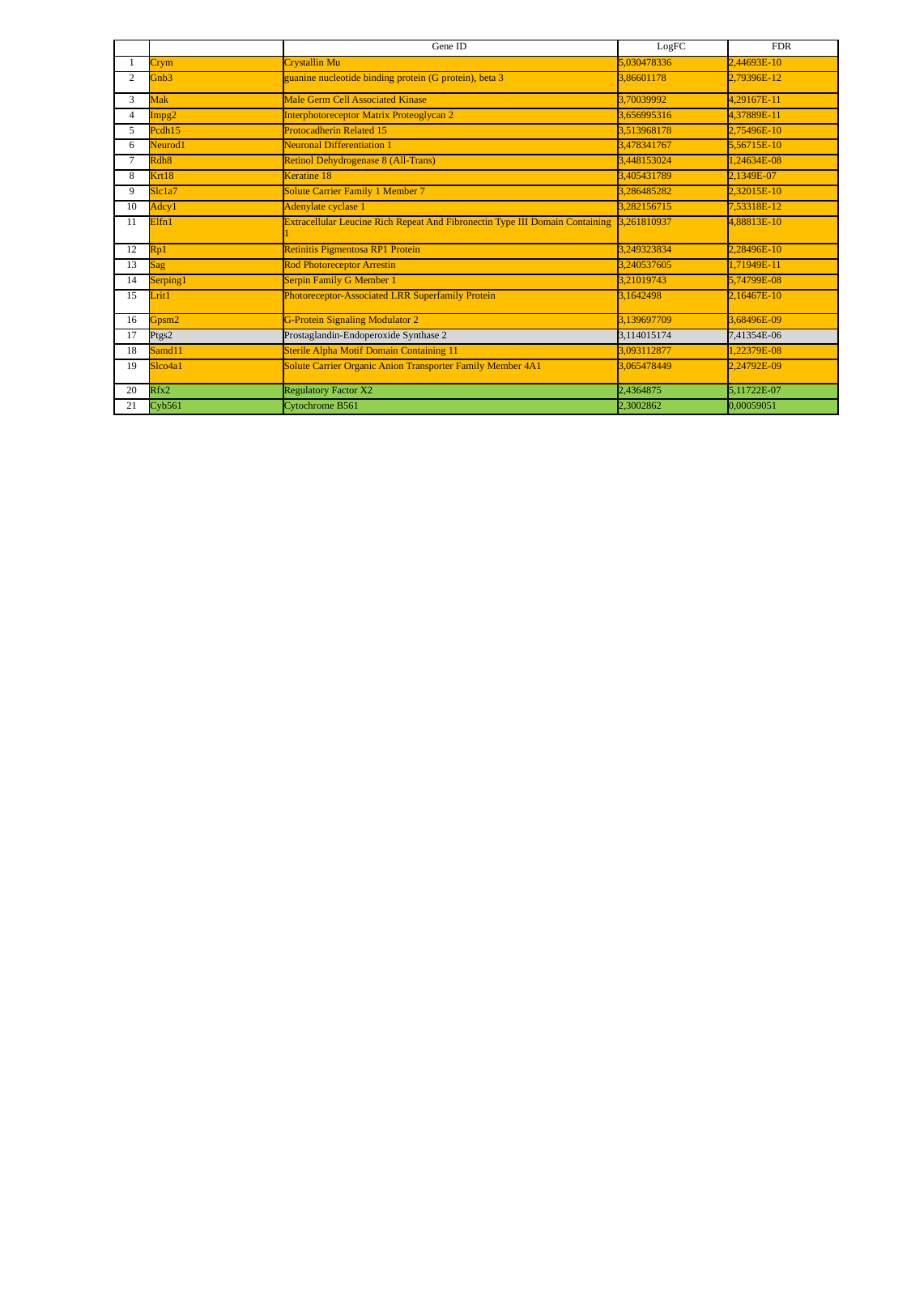

| | | Gene ID | LogFC | FDR |
| --- | --- | --- | --- | --- |
| 1 | Crym | Crystallin Mu | 5,030478336 | 2,44693E-10 |
| 2 | Gnb3 | guanine nucleotide binding protein (G protein), beta 3 | 3,86601178 | 2,79396E-12 |
| 3 | Mak | Male Germ Cell Associated Kinase | 3,70039992 | 4,29167E-11 |
| 4 | Impg2 | Interphotoreceptor Matrix Proteoglycan 2 | 3,656995316 | 4,37889E-11 |
| 5 | Pcdh15 | Protocadherin Related 15 | 3,513968178 | 2,75496E-10 |
| 6 | Neurod1 | Neuronal Differentiation 1 | 3,478341767 | 5,56715E-10 |
| 7 | Rdh8 | Retinol Dehydrogenase 8 (All-Trans) | 3,448153024 | 1,24634E-08 |
| 8 | Krt18 | Keratine 18 | 3,405431789 | 2,1349E-07 |
| 9 | Slc1a7 | Solute Carrier Family 1 Member 7 | 3,286485282 | 2,32015E-10 |
| 10 | Adcy1 | Adenylate cyclase 1 | 3,282156715 | 7,53318E-12 |
| 11 | Elfn1 | Extracellular Leucine Rich Repeat And Fibronectin Type III Domain Containing 1 | 3,261810937 | 4,88813E-10 |
| 12 | Rp1 | Retinitis Pigmentosa RP1 Protein | 3,249323834 | 2,28496E-10 |
| 13 | Sag | Rod Photoreceptor Arrestin | 3,240537605 | 1,71949E-11 |
| 14 | Serping1 | Serpin Family G Member 1 | 3,21019743 | 5,74799E-08 |
| 15 | Lrit1 | Photoreceptor-Associated LRR Superfamily Protein | 3,1642498 | 2,16467E-10 |
| 16 | Gpsm2 | G-Protein Signaling Modulator 2 | 3,139697709 | 3,68496E-09 |
| 17 | Ptgs2 | Prostaglandin-Endoperoxide Synthase 2 | 3,114015174 | 7,41354E-06 |
| 18 | Samd11 | Sterile Alpha Motif Domain Containing 11 | 3,093112877 | 1,22379E-08 |
| 19 | Slco4a1 | Solute Carrier Organic Anion Transporter Family Member 4A1 | 3,065478449 | 2,24792E-09 |
| 20 | Rfx2 | Regulatory Factor X2 | 2,4364875 | 5,11722E-07 |
| 21 | Cyb561 | Cytochrome B561 | 2,3002862 | 0,00059051 |
