# Supplementary material for: Retinal endothelial cell phenotypic modifications during experimental autoimmune uveitis: a transcriptomic approach
Source: BMC Ophthalmol. 2020 Mar 17;20:106. doi: 10.1186/s12886-020-1333-5 (PMC7076950; doi:10.1186/s12886-020-1333-5)
Supplement: Supplementary file 8 — Additional file 8. List of the 82 candidate genes. 82 candidate genes were chosen based on the 2 selection strategies (by variance and/or by expression profile) and ranked by foldchange. Genes in grey correspond to those selected through both analyses. Genes in green were identified through the analysis by expression profile and those in orange through the analysis by variance. [file 12886_2020_1333_MOESM8_ESM.pptx]

## Slide 1
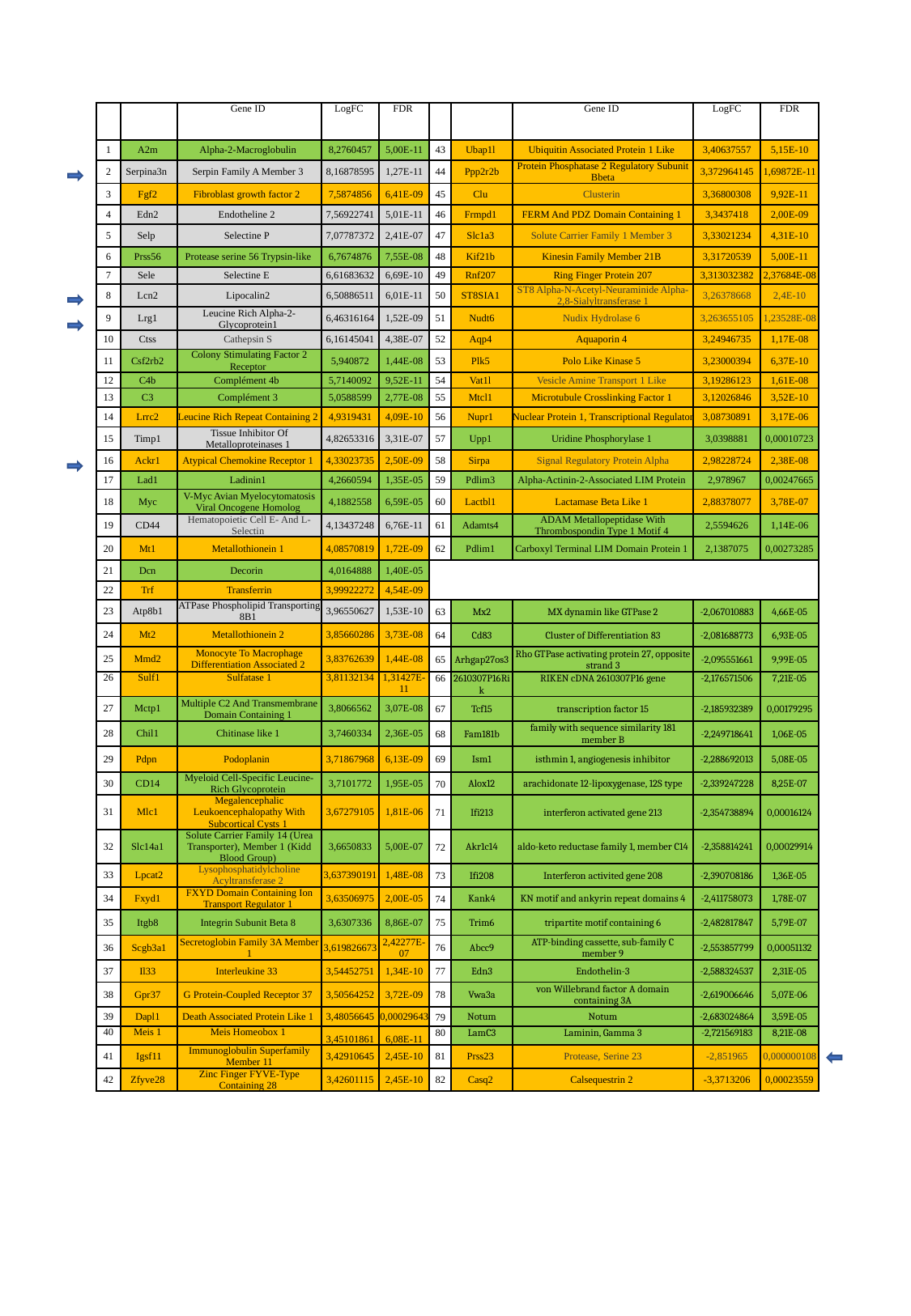

| | | Gene ID | LogFC | FDR | | | Gene ID | LogFC | FDR |
| --- | --- | --- | --- | --- | --- | --- | --- | --- | --- |
| 1 | A2m | Alpha-2-Macroglobulin | 8,2760457 | 5,00E-11 | 43 | Ubap1l | Ubiquitin Associated Protein 1 Like | 3,40637557 | 5,15E-10 |
| 2 | Serpina3n | Serpin Family A Member 3 | 8,16878595 | 1,27E-11 | 44 | Ppp2r2b | Protein Phosphatase 2 Regulatory Subunit Bbeta | 3,372964145 | 1,69872E-11 |
| 3 | Fgf2 | Fibroblast growth factor 2 | 7,5874856 | 6,41E-09 | 45 | Clu | Clusterin | 3,36800308 | 9,92E-11 |
| 4 | Edn2 | Endotheline 2 | 7,56922741 | 5,01E-11 | 46 | Frmpd1 | FERM And PDZ Domain Containing 1 | 3,3437418 | 2,00E-09 |
| 5 | Selp | Selectine P | 7,07787372 | 2,41E-07 | 47 | Slc1a3 | Solute Carrier Family 1 Member 3 | 3,33021234 | 4,31E-10 |
| 6 | Prss56 | Protease serine 56 Trypsin-like | 6,7674876 | 7,55E-08 | 48 | Kif21b | Kinesin Family Member 21B | 3,31720539 | 5,00E-11 |
| 7 | Sele | Selectine E | 6,61683632 | 6,69E-10 | 49 | Rnf207 | Ring Finger Protein 207 | 3,313032382 | 2,37684E-08 |
| 8 | Lcn2 | Lipocalin2 | 6,50886511 | 6,01E-11 | 50 | ST8SIA1 | ST8 Alpha-N-Acetyl-Neuraminide Alpha-2,8-Sialyltransferase 1 | 3,26378668 | 2,4E-10 |
| 9 | Lrg1 | Leucine Rich Alpha-2-Glycoprotein1 | 6,46316164 | 1,52E-09 | 51 | Nudt6 | Nudix Hydrolase 6 | 3,263655105 | 1,23528E-08 |
| 10 | Ctss | Cathepsin S | 6,16145041 | 4,38E-07 | 52 | Aqp4 | Aquaporin 4 | 3,24946735 | 1,17E-08 |
| 11 | Csf2rb2 | Colony Stimulating Factor 2 Receptor | 5,940872 | 1,44E-08 | 53 | Plk5 | Polo Like Kinase 5 | 3,23000394 | 6,37E-10 |
| 12 | C4b | Complément 4b | 5,7140092 | 9,52E-11 | 54 | Vat1l | Vesicle Amine Transport 1 Like | 3,19286123 | 1,61E-08 |
| 13 | C3 | Complément 3 | 5,0588599 | 2,77E-08 | 55 | Mtcl1 | Microtubule Crosslinking Factor 1 | 3,12026846 | 3,52E-10 |
| 14 | Lrrc2 | Leucine Rich Repeat Containing 2 | 4,9319431 | 4,09E-10 | 56 | Nupr1 | Nuclear Protein 1, Transcriptional Regulator | 3,08730891 | 3,17E-06 |
| 15 | Timp1 | Tissue Inhibitor Of Metalloproteinases 1 | 4,82653316 | 3,31E-07 | 57 | Upp1 | Uridine Phosphorylase 1 | 3,0398881 | 0,00010723 |
| 16 | Ackr1 | Atypical Chemokine Receptor 1 | 4,33023735 | 2,50E-09 | 58 | Sirpa | Signal Regulatory Protein Alpha | 2,98228724 | 2,38E-08 |
| 17 | Lad1 | Ladinin1 | 4,2660594 | 1,35E-05 | 59 | Pdlim3 | Alpha-Actinin-2-Associated LIM Protein | 2,978967 | 0,00247665 |
| 18 | Myc | V-Myc Avian Myelocytomatosis Viral Oncogene Homolog | 4,1882558 | 6,59E-05 | 60 | Lactbl1 | Lactamase Beta Like 1 | 2,88378077 | 3,78E-07 |
| 19 | CD44 | Hematopoietic Cell E- And L-Selectin | 4,13437248 | 6,76E-11 | 61 | Adamts4 | ADAM Metallopeptidase With Thrombospondin Type 1 Motif 4 | 2,5594626 | 1,14E-06 |
| 20 | Mt1 | Metallothionein 1 | 4,08570819 | 1,72E-09 | 62 | Pdlim1 | Carboxyl Terminal LIM Domain Protein 1 | 2,1387075 | 0,00273285 |
| 21 | Dcn | Decorin | 4,0164888 | 1,40E-05 | | | | | |
| 22 | Trf | Transferrin | 3,99922272 | 4,54E-09 | | | | | |
| 23 | Atp8b1 | ATPase Phospholipid Transporting 8B1 | 3,96550627 | 1,53E-10 | 63 | Mx2 | MX dynamin like GTPase 2 | -2,067010883 | 4,66E-05 |
| 24 | Mt2 | Metallothionein 2 | 3,85660286 | 3,73E-08 | 64 | Cd83 | Cluster of Differentiation 83 | -2,081688773 | 6,93E-05 |
| 25 | Mmd2 | Monocyte To Macrophage Differentiation Associated 2 | 3,83762639 | 1,44E-08 | 65 | Arhgap27os3 | Rho GTPase activating protein 27, opposite strand 3 | -2,095551661 | 9,99E-05 |
| 26 | Sulf1 | Sulfatase 1 | 3,81132134 | 1,31427E-11 | 66 | 2610307P16Rik | RIKEN cDNA 2610307P16 gene | -2,176571506 | 7,21E-05 |
| 27 | Mctp1 | Multiple C2 And Transmembrane Domain Containing 1 | 3,8066562 | 3,07E-08 | 67 | Tcf15 | transcription factor 15 | -2,185932389 | 0,00179295 |
| 28 | Chil1 | Chitinase like 1 | 3,7460334 | 2,36E-05 | 68 | Fam181b | family with sequence similarity 181 member B | -2,249718641 | 1,06E-05 |
| 29 | Pdpn | Podoplanin | 3,71867968 | 6,13E-09 | 69 | Ism1 | isthmin 1, angiogenesis inhibitor | -2,288692013 | 5,08E-05 |
| 30 | CD14 | Myeloid Cell-Specific Leucine-Rich Glycoprotein | 3,7101772 | 1,95E-05 | 70 | Alox12 | arachidonate 12-lipoxygenase, 12S type | -2,339247228 | 8,25E-07 |
| 31 | Mlc1 | Megalencephalic Leukoencephalopathy With Subcortical Cysts 1 | 3,67279105 | 1,81E-06 | 71 | Ifi213 | interferon activated gene 213 | -2,354738894 | 0,00016124 |
| 32 | Slc14a1 | Solute Carrier Family 14 (Urea Transporter), Member 1 (Kidd Blood Group) | 3,6650833 | 5,00E-07 | 72 | Akr1c14 | aldo-keto reductase family 1, member C14 | -2,358814241 | 0,00029914 |
| 33 | Lpcat2 | Lysophosphatidylcholine Acyltransferase 2 | 3,637390191 | 1,48E-08 | 73 | Ifi208 | Interferon activited gene 208 | -2,390708186 | 1,36E-05 |
| 34 | Fxyd1 | FXYD Domain Containing Ion Transport Regulator 1 | 3,63506975 | 2,00E-05 | 74 | Kank4 | KN motif and ankyrin repeat domains 4 | -2,411758073 | 1,78E-07 |
| 35 | Itgb8 | Integrin Subunit Beta 8 | 3,6307336 | 8,86E-07 | 75 | Trim6 | tripartite motif containing 6 | -2,482817847 | 5,79E-07 |
| 36 | Scgb3a1 | Secretoglobin Family 3A Member 1 | 3,619826673 | 2,42277E-07 | 76 | Abcc9 | ATP-binding cassette, sub-family C member 9 | -2,553857799 | 0,00051132 |
| 37 | Il33 | Interleukine 33 | 3,54452751 | 1,34E-10 | 77 | Edn3 | Endothelin-3 | -2,588324537 | 2,31E-05 |
| 38 | Gpr37 | G Protein-Coupled Receptor 37 | 3,50564252 | 3,72E-09 | 78 | Vwa3a | von Willebrand factor A domain containing 3A | -2,619006646 | 5,07E-06 |
| 39 | Dapl1 | Death Associated Protein Like 1 | 3,48056645 | 0,00029643 | 79 | Notum | Notum | -2,683024864 | 3,59E-05 |
| 40 | Meis 1 | Meis Homeobox 1 | 3,45101861 | 6,08E-11 | 80 | LamC3 | Laminin, Gamma 3 | -2,721569183 | 8,21E-08 |
| 41 | Igsf11 | Immunoglobulin Superfamily Member 11 | 3,42910645 | 2,45E-10 | 81 | Prss23 | Protease, Serine 23 | -2,851965 | 0,000000108 |
| 42 | Zfyve28 | Zinc Finger FYVE-Type Containing 28 | 3,42601115 | 2,45E-10 | 82 | Casq2 | Calsequestrin 2 | -3,3713206 | 0,00023559 |
